# GROMACS in the Cloud: A Global Supercomputer to Speed Up Alchemical Drug Design

**DOI:** 10.1021/acs.jcim.2c00044

**Published:** 2022-03-30

**Authors:** Carsten Kutzner, Christian Kniep, Austin Cherian, Ludvig Nordstrom, Helmut Grubmüller, Bert L. de Groot, Vytautas Gapsys

**Affiliations:** † Department of Theoretical and Computational Biophysics, Max Planck Institute for Multidisciplinary Sciences, Am Fassberg 11, 37077 Göttingen, Germany; ‡ Amazon Development Center Germany, Amazon Web Services, Krausenstr. 38, 10117 Berlin, Germany; ¶ Amazon Web Services Singapore Pte Ltd, 23 Church St, #10-01, Singapore 049481; § Amazon Web Services, 60 Holborn Viaduct, London EC1A 2FD, United Kingdom; ∥ Computational Biomolecular Dynamics Group, Max Planck Institute for Multidisciplinary Sciences, Am Fassberg 11, 37077 Göttingen, Germany

## Abstract

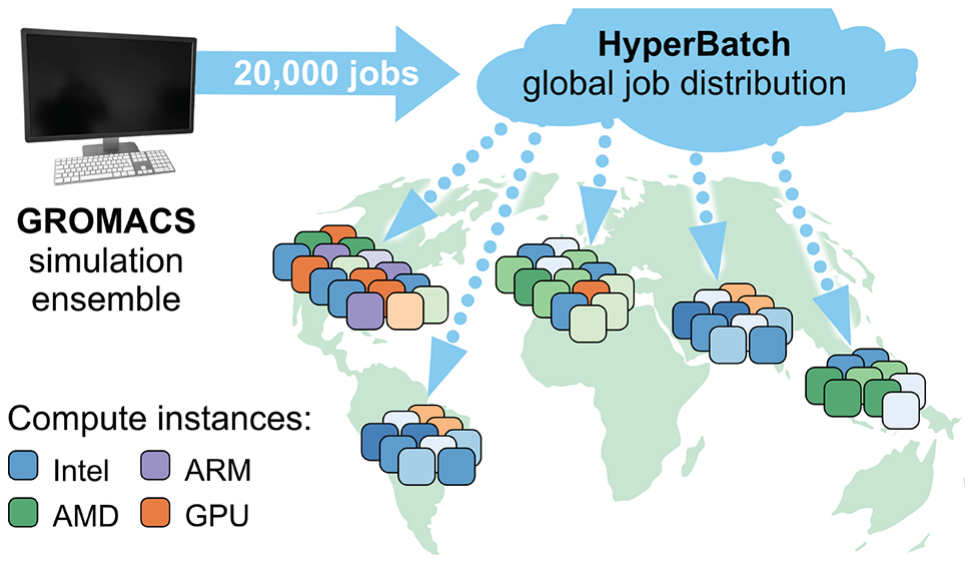

We assess costs and efficiency of state-of-the-art high-performance cloud computing and compare the results to traditional on-premises compute clusters. Our use case is atomistic simulations carried out with the GROMACS molecular dynamics (MD) toolkit with a particular focus on alchemical protein–ligand binding free energy calculations. We set up a compute cluster in the Amazon Web Services (AWS) cloud that incorporates various different instances with Intel, AMD, and ARM CPUs, some with GPU acceleration. Using representative biomolecular simulation systems, we benchmark how GROMACS performs on individual instances and across multiple instances. Thereby we assess which instances deliver the highest performance and which are the most cost-efficient ones for our use case. We find that, in terms of total costs, including hardware, personnel, room, energy, and cooling, producing MD trajectories in the cloud can be about as cost-efficient as an on-premises cluster given that optimal cloud instances are chosen. Further, we find that high-throughput ligand-screening can be accelerated dramatically by using global cloud resources. For a ligand screening study consisting of 19 872 independent simulations or ∼200 μs of combined simulation trajectory, we made use of diverse hardware available in the cloud at the time of the study. The computations scaled-up to reach peak performance using more than 4 000 instances, 140 000 cores, and 3 000 GPUs simultaneously. Our simulation ensemble finished in about 2 days in the cloud, while weeks would be required to complete the task on a typical on-premises cluster consisting of several hundred nodes.

## 1 Introduction

Over the past decades, molecular dynamics (MD) simulations have become a standard tool to study biomolecules in atomic detail. In the field of rational drug design, MD can greatly reduce costs by transferring parts of the laboratory workflow to the computer. In the early stage of drug discovery, large libraries of small molecules with the potential to bind to the target protein (the “hits”) are identified and subsequently modified and optimized to ultimately uncover more potent “lead” candidates. *In silico* approaches allow reducing the number of small molecule compounds from tens of thousands down to a few hundred entering preclinical studies.

Naturally, it is a combination of all the pharmacokinetic and pharmacodynamic features that defines whether a candidate molecule can be evolved into a useful drug. Molecular dynamics-based computational drug development concentrates mainly on the particular question of how well a specific ligand binds to a target. While calculations of absolute protein–ligand binding affinity are feasible, they also present numerous technical challenges.^1,2^ Evaluation of the relative binding affinities, however, is much more tractable and in recent years has been well-established in the field of computational chemistry.^3−6^ In the latter approach, MD-based so-called *alchemical* calculations allow obtaining differences in binding free energy between two ligands. Such calculations require performing transformations between the two ligands for their protein-bound and for their unbound solvated state. Carrying out multiple transformations allows sorting the whole collection of ligands by their binding affinity to the target. Different approaches can be used to carry out the transformations, but they all involve a λ parameter that interpolates between the ligands. An automated workflow for binding affinity calculations has recently been developed,^5^ based on the open-source software packages pmx^7^ and GROMACS.^8^

Despite continuous advances in hardware and software, carrying out MD simulations remains computationally challenging. A typical MD project can easily occupy a modern compute cluster for days or even months until a sufficient amount of simulation trajectory is produced.

Where now does a researcher get the required compute time? Established providers are the compute centers of universities and research institutes, national supercomputing centers, and local clusters, each with particular advantages and disadvantages with respect to how easily resources can be accessed, how much and how quickly they are available, what the costs are, *etc*. During the past decade, cloud computing^9,10^ has developed into a new, alternative option to obtain compute time for scientific applications.

Whereas the first systems of cloud computing reach back into the mid-1990s,^11^ since about 2007, it is being increasingly used for scientific workloads.^12−16^ Cloud computing providers like Amazon Web Services (AWS), Microsoft Azure, or Google Cloud Platform can serve both HPC and HTC demands as they nowadays offer virtually limitless compute power, plus the possibility to efficiently parallelize individual simulations over multiple instances (compute nodes in the cloud) connected by a high-performance network.

One of the main promises of cloud-based computing is its ability to easily scale-up when the resources for computation are required. This way the user has access to an HPC/HTC facility which can flexibly adjust to the particular needs at a given time. Consequently, the usage of such cloud compute clusters can be fine-tuned to optimize costs or minimize the time-to-solution.

The Folding@home project, initiated in 2000, was the first to leverage globally distributed compute resources for MD on a large scale.^17^ Reports of cloud infrastructure in the narrow sense being used for MD date back to 2012. Wong *et al*. developed a VMD^18^ plugin for the NAMD^19^ simulation software that simplifies running simulations in the AWS Elastic Compute Cloud (EC2).^20^ They carried out a simulation of a one million atom large biomolecular system on a total of 64 CPU cores spread over eight EC2 instances. Van Dijk *et al*. implemented a web portal to execute large-scale parametric MD studies with GROMACS^8^ on European grid resources.^21^ In 2014, Król *et al*. performed an ensemble simulation of 240 replicas of a several hundred atom large evaporating nanodroplet on 40 EC2 single-core instances.^22^ Kohlhoff *et al*. demonstrated that long simulation time scales for biomolecules can be accessed with the help of cloud computing. They simulated two milliseconds of dynamics of a major drug target on the Google Exacycle platform.^23^ Singharoy *et al*. described tools to easily perform MD-based flexible fitting of proteins into cryo-EM maps in the AWS cloud.^24^

The concept of making cloud-based workflows for MD readily available to the scientist is also pursued by the following projects. The AceCloud^25^ on-demand service facilitates running large ensembles of MD simulations in the AWS cloud; it works with the ACEMD,^26^ GROMACS, NAMD, and Amber^27^ simulation packages. QwikMD^28^ is a user-friendly general MD program integrated into VMD and NAMD that runs on supercomputers or in the AWS cloud. HTMD^29^ is a python-based extensible toolkit for setting up, running, and analyzing MD simulations that also comes with an AWS interface. A purely web-based application that facilitates setting up MD simulations and running them in the cloud is described by Nicolas-Barreales *et al*.^30^ The Copernicus^31^ scientific computing platform can be used to carry out large sets of MD simulations on supercomputers and cloud instances. In a hands-on fashion, Kohlhoff describes how to perform GROMACS simulations on Google’s cloud platform using Docker containers.^32^ Arantes *et al*. propose a Jupyter-notebook based, user-friendly solution to perform different kinds of MD-related workflows at no cost using the Google Colab services, which is especially useful for teaching purposes.^33^

Cloud computing is also increasingly being adopted to aid drug discovery. In their 2013 article,^34^ Ebejer *et al*. review the use of cloud resources for protein folding and virtual screening and highlight the potential for future large-scale, data-intensive molecular modeling applications. They point out a virtual screening study of 21 million compounds that has been carried out in 2012 on a cloud-based cluster with 50 000 cores using Schrödinger’s docking software Glide.^35^ D’Agostino *et al*. discuss the economic benefits of moving *in silico* drug discovery workflows to the cloud.^36^ A recent virtual screening study of 1 billion compounds against proteins involved in SARS-CoV-2 infection was carried out on Google’s cloud services.^37^

Cloud computing has been compared to traditional on-premises clusters for exemplary scientific workflows;^38,39^ however, we are unaware of a quantitative study to date for the field of MD simulation. Therefore, here we assess the costs and performance of cloud computing for carrying out biomolecular simulations. We use GROMACS as the simulation engine and AWS as the provider of cloud infrastructure for this case study, for the following reasons: GROMACS is open source and freely available to anyone, and it is one of the fastest MD codes available.^40^ AWS is one of the largest providers of cloud infrastructure, on par with Microsoft and Google.^10^

First, we measured the GROMACS performance using established benchmarks^41^ on a broad range of available instance types (with and without GPUs) and also across multiple instances. The simulation performance-to-instance price ratio allows optimizing for a minimal time-to-solution or minimal project costs. The benchmark results and the instance costs allowed us to compare the costs of carrying out simulations in the cloud to those for operating an in-house cluster. Second, we ask how much high-throughput ligand screening can be accelerated in the cloud. To address this question, we used globally available compute capacity to carry out a large protein–ligand binding affinity study at highest possible throughput.

## 2 General Background

### 2.1 Cloud Computing

The large cloud providers offer a wide range of instance types, with and without GPUs, optionally with extra memory or HPC network, targeted toward different application areas. Because of the sheer scale of options, cloud resources can appear overwhelming at first, especially compared to an on-premises HPC cluster with preinstalled software. To help setting up typical workflows and to illustrate best practices, plenty of online content is available such as manuals, tutorials, workshops and discussion forums. In addition, an experienced technical staff is available to directly help with specific issues—a support that this project has greatly benefited from.

The compute unit that is rented out to customers is called *instance*. It may be a whole node with multiple cores and GPU(s), just a part of a node, or even just a single core.

Large nodes that are rented out as several smaller instances are shared between different customers. However, each customer is restricted to her instance (her part of the node) exclusively, and her processes cannot spill over into the compute cores, memory, or network bandwidth allocated to other instances on the node. AWS instances come with a certain number of virtual CPUs (vCPUs) which translate to hardware threads. Renting two vCPUs on a modern AMD or Intel-based instance is equivalent to getting one physical core exclusively on that machine.

Although the actual exact location of allocated compute instances remains opaque to the user, the *region* she chooses encompasses a group of geographically close data centers. Costs usually vary by region, depending on supply and demand, as well as energy costs, and specific services or cutting edge processor features may be available only in some of the regions. For the case of AWS, each region consists of multiple, isolated, and physically separate *availability zones* (AZs) within a geographic area. An AZ is a group of one or more data centers with independent redundant power supply and network connectivity. In 2021, AWS had 85 AZs in 26 regions.

There are different payment models that can be chosen from. *On-demand* payment is most flexible, as one can rent an instance at any time and give it back when it is not needed any more. One pays only for the time that the instance is needed. One can also get *reserved instances* at a 50–70% discount if one books these instances for one to three years, but then one has to pay regardless if one can make use of them. *Preemptible* or *Spot* instances tap into the pool of currently unused compute capacity and are available at discount rates of up to 90% compared to on-demand, though pricing varies across AZs and over time. However, a Spot instance can be claimed back at any time by Amazon EC2 with a 2 min warning.

### 2.2 Using Hardware Efficiently with GROMACS

Key to optimal simulation performance is understanding how GROMACS makes use of the available hardware. GROMACS combines several parallelization techniques, among them MPI and OpenMP parallelism, GPU offloading, and separable ranks to evaluate long-range electrostatics. With domain decomposition (DD), the simulation system is divided into *n*_*x*_ × *n*_*y*_ × *n*_*z*_ domains, each of which is operated on by one MPI rank.^40^ During the simulation, dynamic load balancing (DLB) adjusts the size of the domains such that any uneven computational load between the MPI ranks is minimized.

Each MPI rank can further have multiple OpenMP threads. Best performance is usually achieved when the product of MPI ranks and OpenMP threads equals the number of cores (or hardware threads) on a node or instance and when all threads are properly pinned to cores. Though leaving some cores idle may in rare cases make sense, oversubscription will lead to significant performance degradation.

When distributing a simulation system over an increasing number of MPI ranks in a strong scaling scenario, at some point the time spent for communication between the ranks limits further speedup. Usually the bottleneck is in the long-range contribution to the electrostatic forces which are calculated with the particle mesh Ewald (PME) method.^42^ Parallel PME requires all-to-all communication between the participating ranks, leading to *r*^2^ MPI messages being sent on *r* MPI ranks.^40^ This communication bottleneck can be alleviated by assigning a subset of MPI ranks to exclusively evaluate the long-range PME part. As typically only a quarter up to a third of all ranks need to be allocated for long-range electrostatics, the communication bottleneck is greatly reduced, yielding better performance and scalability.

GROMACS can offload various types of computationally demanding interactions onto the GPU.^41,43,44^ One of the largest performance benefits stems from offloading the short-range part of the nonbonded interactions (Coulomb and van der Waals). In parallel, each MPI rank can offload its local domain’s interactions to a GPU. The PME long-range part can be offloaded as well; however, this computation still cannot be distributed onto multiple GPUs. Additionally, bonded interactions and for suitable parameter settings the integration and constraint calculations can be offloaded.

The relative GPU-to-CPU compute power on a node determines how many interaction types can be offloaded for optimal performance. Ideally, CPU and GPU finish their force calculation at about the same time in the MD time step so that no time is lost waiting.

Earlier studies showed that both the GROMACS performance as well as the performance-to-price (P/P) ratio, *i.e.*, how much MD trajectory is produced per invested €, can vastly differ for different hardware.^41,45^ Nodes with GPUs provide the highest single-node GROMACS performance. At the same time, P/P skyrockets when consumer GPUs are used instead of professional GPUs (*e.g.*, NVIDIA GeForce RTX instead of Tesla GPUs). The P/P ratio of consumer GPU nodes is typically at least a factor of 3 higher than that of CPU nodes or nodes with professional GPUs.

Pronounced variations in GROMACS performance and cost-efficiency are therefore expected between the different instance types on AWS. Benchmarks allow picking instance types optimal for MD simulation.

### 2.3 Obtaining Relative Binding Free Energies from MD Simulations

To evaluate relative binding affinities in a chemical library of interest, ligands are connected into a network (graph) and a number of pairwise calculations is performed, eventually allowing the sorting of the molecules according to their binding free energy. It is a usual practice to repeat calculations several times for each ligand pair to obtain reliable uncertainty estimates.^46−48^

Various methods for the alchemical calculations have been developed. For example, the commercially available Schrödinger software uses a free energy perturbation-based approach,^49^ whereas the open source workflow used here^5,7^ is based on thermodynamic integration (TI)^50^ using a nonequilibrium transformation protocol.^51^ Both approaches yield similarly accurate relative binding free energies at similar computational effort.^5^

The nonequilibrium TI approach requires equilibrated ensembles of the physical end states for the solvated protein with ligand, one for ligand A and one for ligand B, as well as two equilibrated ensembles of ligand A and ligand B in solution. From the equilibrated ensembles, many short “fast growth” TI simulations are spawned during which ligand A is transformed into ligand B and *vice versa* using a λ-dependent Hamiltonian. The free energy difference is then derived from the overlap of the forward (A → B) and reverse (B → A) work distributions using estimators based on the Crooks fluctuation theorem.^52^

## 3 Methods

We will first describe the setup of the cloud-based HPC clusters that we used to derive the GROMACS performance on a range of available instance types and provide some details about the benchmark input systems and on how the benchmarks were carried out. Then we will outline our setup to distribute a large ensemble of free energy calculations on globally available compute resources.

### 3.1 Cloud-Based HPC Cluster and Software Setup

The benchmark simulations were carried out on AWS compute clusters in the North Virginia region set up with the ParallelCluster^53^ open source cluster management tool. Each cluster consists of a master instance of the same architecture as the nodes (x86 or ARM). The master fires up and closes down the node instances as needed and operates the queueing system (SLURM).^54^ For the x86 cluster, we used ParallelCluster v. 2.10.0 on a c5.2xlarge master; for the ARM cluster, we used v. 2.9.1 on a m6g.medium master instance. For brevity, we will from now on refer to c5.2xlarge instances as c5.2xl and also abbreviate all other *xlarge instances accordingly. All instances use Amazon Linux 2 as operating system; for technical specifications of the instances, see Table 1. Whereas all instances can communicate *via* TCP (see last columns in Table 1 for the network bandwidth), some of them have an elastic fabric adapter (EFA). EFA enables HPC scalability across instances by a higher throughput compared to TCP and a lower and more consistent latency.

**Table 1:** Technical Specifications of AWS Instances Used in This Study and GROMACS Compilation Options^a^

							network	
instance type	CPU model	HT or vCPUs	clock (GHz)	used SIMD instructions	NVIDIA GPUs	MPI lib	(Gbps)	EFA
c5.24xl	Intel 8275CL	96	3.0	AVX_512		i	25	
c5.18xl	Intel 8124M	72	3.0	AVX_512		i	25	
c5n.18xl	Intel 8124M	72	3.0	AVX_512		i	100	*√*
c5.12xl	Intel 8275CL	48	3.0	AVX_512		i	12	
c5.9xl	Intel 8124M	36	3.0	AVX_512		i	10	
c5.4xl	Intel 8275CL	16	3.0	AVX_512		i	≤10	
c5.2xl	Intel 8275CL	8	3.0	AVX_512		i	≤10	
c5.xl	Intel 8275CL	4	3.0	AVX_512		i	≤10	
c5.large	Intel 8124M	2	3.0	AVX_512		i	≤10	
c5a.24xl	AMD EPYC 7R32	96	3.3	AVX2_128		i	20	
c5a.16xl	AMD EPYC 7R32	64	3.3	AVX2_128		i	20	
c5a.12xl	AMD EPYC 7R32	48	3.3	AVX2_128		i	12	
c5a.8xl	AMD EPYC 7R32	32	3.3	AVX2_128		i	10	
c5a.4xl	AMD EPYC 7R32	16	3.3	AVX2_128		i	≤10	
c5a.2xl	AMD EPYC 7R32	8	3.3	AVX2_128		i	≤10	
c5a.xl	AMD EPYC 7R32	4	3.3	AVX2_128		i	≤10	
c5a.large	AMD EPYC 7R32	2	3.3	AVX2_128		i	≤10	
hpc6a.48xl	AMD EPYC 7R13	96	2.65	AVX2_128		t	100	*√*
c6g.16xl	ARM Graviton2	64	2.3	NEON_ASIMD		t	25	
c6g.12xl	ARM Graviton2	48	2.3	NEON_ASIMD		t	20	
c6g.8xl	ARM Graviton2	32	2.3	NEON_ASIMD		t	≤10	
c6g.4xl	ARM Graviton2	16	2.3	NEON_ASIMD		t	≤10	
c6g.2xl	ARM Graviton2	8	2.3	NEON_ASIMD		t	≤10	
c6g.xl	ARM Graviton2	4	2.3	NEON_ASIMD		t	≤10	
c6i.32xl	Intel 8375C	128	2.9	AVX_512		i	50	*√*
m6i.32xl	Intel 8375C	128	2.9	AVX_512		t	50	*√*
m5n.24xl	Intel 8259CL	96	2.5	AVX_512		i	100	*√*
m5zn.12xl	Intel 8252C	48	3.8	AVX_512		t	100	*√*
m5zn.2xl	Intel 8252C	8	3.8	AVX_512		t	≤25	
p3.2xl	Intel E5-2686v4	8	2.3	AVX2_256	V100	t	≤10	
p3.8xl	Intel E5-2686v4	32	2.3	AVX2_256	V100 × 4	t	10	
p3.16xl	Intel E5-2686v4	64	2.3	AVX2_256	V100 × 8	t	25	
p3dn.24xl	Intel 8175M	96	2.5	AVX2_256	V100 × 8	t	100	*√*
p4d.24xl	Intel 8275CL	96	3.0	AVX2_256	A100 × 8	i	400	*√*
g3s.xl	Intel E5-2686v4	4	2.3	AVX2_256	M60	i	10	
g3.4xl	Intel E5-2686v4	16	2.3	AVX2_256	M60	i	≤10	
g4dn.xl	Intel 8259CL	4	2.5	AVX_512	T4	i	≤10	
g4dn.2xl	Intel 8259CL	8	2.5	AVX_512	T4	i	≤25	
g4dn.4xl	Intel 8259CL	16	2.5	AVX_512	T4	i	≤10	
g4dn.8xl	Intel 8259CL	32	2.5	AVX_512	T4	i	50	
g4dn.12xl	Intel 8259CL	48	2.5	AVX_512	T4	i	50	
g4dn.16xl	Intel 8259CL	64	2.5	AVX_512	T4	i	50	
g4dn.12xl	Intel 8259CL	48	2.5	AVX_512	T4 × 4	i	50	
g5.xl	AMD EPYC 7R32	4	3.3	AVX2_128	A10G	t	≤10	
g5.2xl	AMD EPYC 7R32	8	3.3	AVX2_128	A10G	t	≤10	
g5.4xl	AMD EPYC 7R32	16	3.3	AVX2_128	A10G	t	≤25	
g5.8xl	AMD EPYC 7R32	32	3.3	AVX2_128	A10G	t	25	

a i, using Intel MPI 2019; t, using GROMACS’ built-in thread-MPI library. EFA (elastic fabric adapter) signals whether an HPC network is available.

Different versions of GROMACS (2020.2 and 2021.1, with and without MPI) were installed with the Spack^55^ 0.15.4 package manager. GROMACS was built in mixed precision with GCC 7.3.1, FFTW 3.3.8, hwloc 1.11, and either Intel MPI 2019 or its built-in thread-MPI library (as listed in Table 1). GPU versions used CUDA 10.2 on g instances and CUDA 11.1 on p instances. Benchmarks on m6i.32xl instances were done using ICC 2021.2 and Intel MKL. The multi-instance scaling benchmarks on m5n.24xl and m5zn.12xl instances used a GROMACS executable built with Intel MPI + ICC 2021.2 and Intel MKL.

A workshop to reproduce a (slightly updated) setup is available on the web,^56^ whereas general advice on how to use AWS services can be found in this book.^57^

### 3.2 Description of the MD Benchmark Systems

To determine the GROMACS performance on various instance types, we used seven simulation systems (Table 2). MEM, RIB, and PEP are typical MD systems differing in size and composition, where no special functionality like external forces or free energy (FE) is required. MEM is an aquaporin tetramer embedded in a lipid membrane surrounded by water and ions in a simulation box of 10.8 × 10.2 × 9.6 nm^3^ size.^58^ RIB contains an *E. coli* ribosome in a box of size (31.2 nm)^3^ with water and ions.^59^ The (50 nm)^3^ large PEP system was used to study peptide aggregation;^60^ it contains 250 steric zipper peptides in solution. MEM, RIB, and PEP were used in previous performance studies,^41,45,61^ allowing the comparison of cloud instances to a variety of other already benchmarked hardware.

**Table 2:** Benchmark Systems: Specifications of the MD Input Systems That Are Used for Benchmarks in This Study^a^

benchmark acronym	no. of atoms	Δ*t* (fs)	*r*_c_ (nm)	grid sp. (nm)	no. of FE atoms
PEP^61^	12 495 503	2	1.2	0.160	0
RIB^59^	2 136 412	4	1.0	0.135	0
MEM^58^	81 743	2	1.0	0.12	0
SHP-2 protein + ligand	107 330	2	1.1	0.12	53
c-Met protein + ligand	67 291	2	1.1	0.12	61
HIF-2α protein + ligand	35 546	2	1.1	0.12	35
c-Met ligand in water	6 443	2	1.1	0.12	61

a The FE column lists the number of perturbed atoms for this benchmark (note that this number will vary for different ligands considered in the physical end states); Δ*t* is integration time step, *r*_c_ cutoff radius, and grid sp. the spacing of the PME grid. Benchmark input .tpr files can be downloaded from https://www.mpinat.mpg.de/grubmueller/bench.

c-Met, HIF-2α, and SHP-2 are representative systems from the large binding affinity ensemble assembled by Schindler *et al*.^4^ These systems run special FE kernels for all λ-dependent interactions, *i.e.*, those involving a transformation between atomic properties. As the FE kernels are slower than the normal kernels and because of a larger cutoff, finer PME grid, and the need to calculate two PME grids (one for each of the physical states), even at equal atom count a FE simulation will be slower than a plain MD system. We therefore carried out separate benchmarks for the FE systems, chosen such that predicting the performance of all ensemble members listed in Tables 3 and 4 is easily possible: A small, medium, and large protein plus ligand system to cover the whole range of sizes for the protein systems (35 k–110 k atoms) and one ligand-in-water system representative for all 9936 ligand-in-water simulations.

**Table 3:** Systems Considered for the First Binding Affinity Study^a^

	size (atoms)				
system	protein+ligand	ligand	no. of ligands	no. of edges	no. of jobs
CDK8	109 807	5789	33	54	972
SHP-2	107 330	6231	26	56	1008
PFKFB3	96 049	6570	40	67	1206
Eg5	79 653	6116	28	65	1170
c-Met	67 291	6443	24	57	1026
SYK	66 184	5963	44	101	1818
TNKS2	52 251	6012	27	60	1080
HIF-2α	35 546	4959	42	92	1656
total					2 × 9936

a For each of eight considered protein–ligand complexes (from the study^4^), two sets of simulations are performed: *protein+ligand* for the solvated protein–ligand complex and *ligand* for the solvated ligand alone. An *edge* is referred to as the transformation of one ligand A to another ligand B. As we probe three independent replicas for each system in forward and backward simulation direction, and three small molecule force fields (GAFF^62^ v2.11, CGenFF^63,64^ v3.0.1, and OpenFF^65^ v2.0.0), the total number of jobs is 3 × 2 × 3 = 18× the number of edges for the *protein+ligand* plus an equal number for the *ligand* systems.

**Table 4:** Systems Considered for the Second Binding Affinity Study^a^

	size (atoms)				
system	protein+ligand	ligand	no. of ligands	no. of edges	no. of jobs
CDK2	106 910	4993	16	25	150
P38	80 777	6750	34	56	336
ROS1	73 957	8434	28	63	378
Bace	73 330	5914	36	58	348
JNK1	72 959	5956	21	31	186
Bace (Hunt)	72 036	5773	32	60	360
Bace (p2)	71 671	6687	12	26	156
PTP1B	70 020	8753	23	49	294
PDE2	63 943	5504	21	34	204
TYK2	62 292	5956	16	24	144
PDE10	56 616	7655	35	62	372
thrombin	49 312	6025	11	16	96
galectin	35 635	9576	8	7	42
MCL1	32 745	5435	42	71	426
total					2 × 3492

a Same as in Table 3, but considering 14 protein–ligand complexes in one MD force field (OpenFF v2.0.0). The systems were collected from public sources for the previous free energy calculation studies.^5,66^ The total number of jobs is 3 × 2 = 6× the number of edges for the *protein+ligand* plus an equal number for the *ligand* systems.

In total, 2 × 9 936 = 19 872 independent jobs were run for the binding affinity study (Table 3) by which 1656 free energy differences (ΔΔ*G* values) were determined. Each job first simulated six nanoseconds at equilibrium (for the starting state, *i.e.*, A or B), followed by 80 nonequilibrium transformations from the start to the end state (A → B, or B → A), as mentioned in section 2.3. The 80 individual transformations were started from different, equidistant positions of the equilibrium trajectory and were each 50 ps long. In total, 10 ns of trajectory was generated per job.

### 3.3 Benchmarking Procedure

#### 3.3.1 MEM and RIB Plain MD Systems

MEM and RIB benchmarks were run for 20 k steps on single instances and for 40 k–50 k steps when scaling across multiple instances or multiple GPUs using GROMACS 2020. Because of effects of load balancing, PME grid versus cutoff scaling and memory allocations (compare section 2.2) the first few thousand steps in a GROMACS simulation are typically slower than average and were therefore excluded from the benchmarks, which are intended to be proxies for the long-term performance.

To make use of all CPU cores of an instance, the product of ranks × threads was set to the number of physical cores or to the number of available hardware threads. We benchmarked various combinations of ranks × threads and additionally checked whether separate PME ranks improve performance. Pinning of threads to cores was enabled, and no checkpoint files were written during the benchmark runs.

On GPU instances we used one rank per GPU and offloaded all short-range nonbonbed interactions to the GPU(s). For improved performance, also the long-range PME contribution was offloaded to a GPU, except for some GPU instances with many cores, where it turned out to be faster to evaluate the long-range PME contribution on the CPU. For scaling benchmarks across two or more GPU instances, the long-range PME contribution was run on the CPU part, as only there can it be parallelized.

The timings (in simulated nanoseconds per day) reported for MEM and RIB (Tables 5–9) are averages over two runs. The parallel efficiency on *n* instances *E*_*n*_ reported in Tables 7, 8, and 9 is computed as the performance *P*_*n*_ on *n* instances divided by *n* times the performance on a single instance:
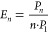
1The performance-to-price ratios (ns/$) in the MEM and RIB tables are calculated from Amazon EC2 on-demand prices for Linux instance in the US East (N. Virginia) region (https://aws.amazon.com/ec2/pricing/on-demand/), except for hpc6a instances, which are not yet available in N. Virginia. Therefore, for those instances, the prices in the US East (Ohio) region were used.

#### 3.3.2 Free Energy Systems Used for the Binding Affinity Study

Each job of the binding affinity ensemble run (Table 2) consists of two parts: first, a 6 ns equilibrium simulation; second, 80 nonequilibrium transformations of 50 ps length each.

The first (equilibration) part was benchmarked as described above for MEM and RIB, using 10 k total steps with timings from the first half discarded. In cases where PME grid versus cutoff tuning took more than 5 k time steps, 20 k time steps were used in total. For the binding affinity runs we did not check whether separate PME ranks improve the performance. The timings reported in Tables 10 and 11 resulted from individual runs of the equilibration part. Here, we ran on individual instances only, no scaling across multiple instances was attempted. Though in most cases we tested various combinations of splitting a given number of total cores *N*_c_ into ranks and threads *N*_c_ = *N*_ranks_ × *N*_threads_, we do not report all results in Tables 10 and 11 to keep them reasonably concise. Instead, we report a consensus for the combination *N*_ranks_ × *N*_threads_ that yielded best results across the free energy benchmark systems.

The second (transformation) part was benchmarked by timing one of the 50 ps (25 k steps) long transformation runs. No time steps were discarded from the measurements, as an initial below-average performance will occur in each of the 80 short transformation runs and thus should be included when predicting the performance of the whole transformation part.

The total costs per free energy difference have been derived by combining the equilibration and transformation phase timings of the protein–ligand complex and the ligand alone in water. Six runs were performed per free energy difference for the protein–ligand complex (3 replicas × 2 directions) plus additional six for the solvated ligand. All runs for the solvated ligand were performed on c5.2xl instances. As the 12 independent parts ran in parallel, the time-to-solution is given by the runtime of the longest individual part. That would usually be the protein–ligand complex, but in rare cases the ligand in water would have a longer runtime, as it ran on comparatively slow c5.2xl instances. Prices for AWS instances in the US East (N. Virginia) region as of May 2021 were used.

### 3.4 Setup of Globally Distributed Compute Resources

The allocation of cloud-based compute resources (*e.g.*, *via* ParallelCluster or AWS Batch^67^) is normally confined to a specific geographic region (there are currently 26 in AWS). Whereas stacks of small to medium jobs can be conveniently executed using just a single region, a global setup is better suited when a substantial amount of core hours is needed: The pool of available instances is much larger for all regions combined compared to just a single region. This allows, for example, to start more instances at the same time or to pick only the subset of instances with the highest performance-to-price ratio. To benefit from global compute resources, we used AWS HyperBatch as a means to provide a single entry point for jobs scheduled to AWS Batch queues across regions.

**Figure 1:**
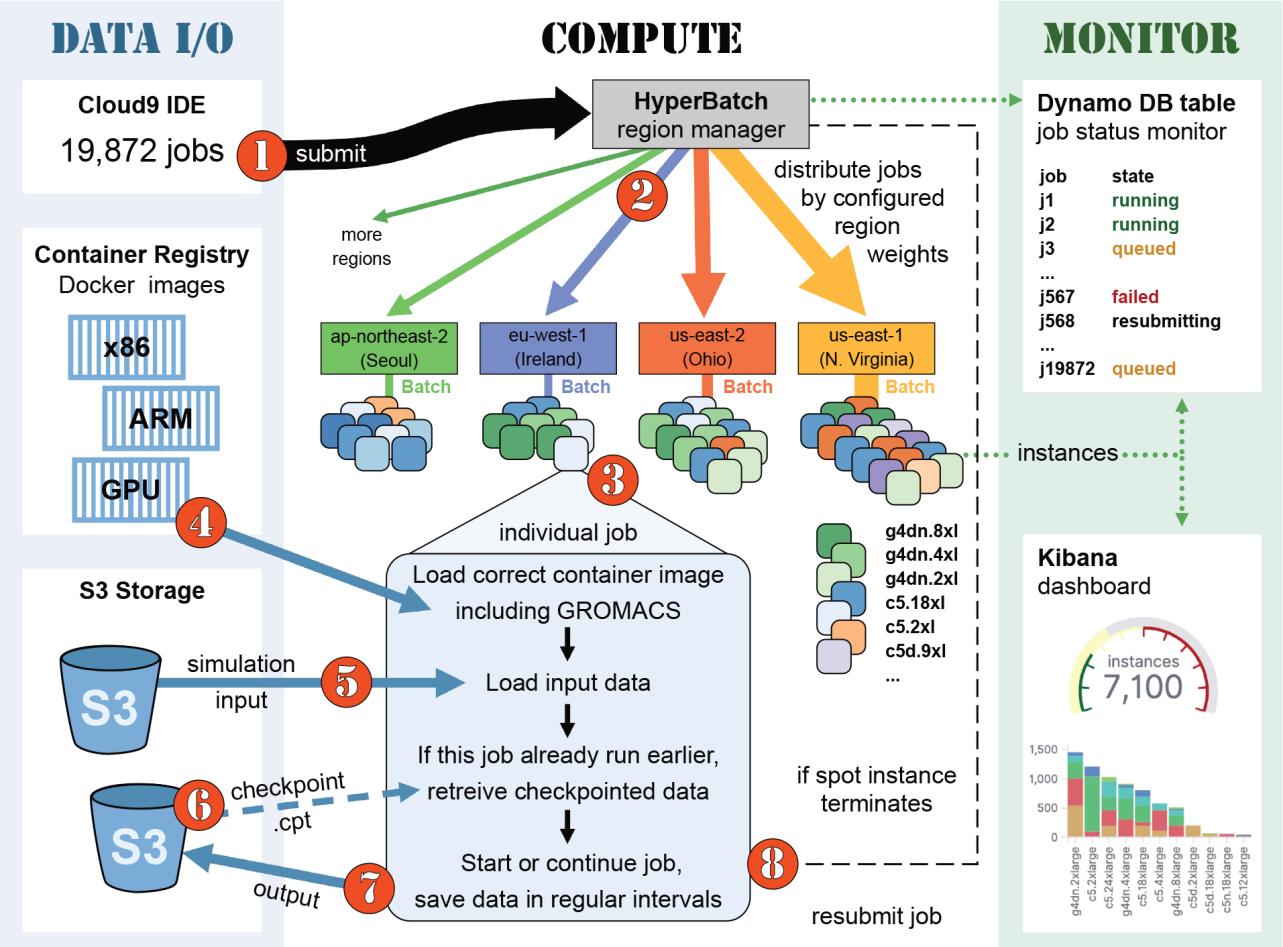
HyperBatch-based setup distributes all 19 872 GROMACS jobs globally. An illustrative lifetime of a job follows the steps ①···⑧ and is described in section 3.4 of the text.

The technical setup used for the binding affinity study is sketched in Figure 1. For easier overview, the main compute setup is shown in the middle, whereas input and output of data is gathered in the left, blue column and monitoring functionality about the status of jobs and instances in the right, green column. In a nutshell, AWS HyperBatch provides cross-regional serverless job scheduling and resource orchestration using DynamoDB, Lambda functions, Step Functions, AWS Kinesis Data Streams, the Simple Queue Service (SQS), and the Amazon API Gateway.^57^

**Figure 2:**
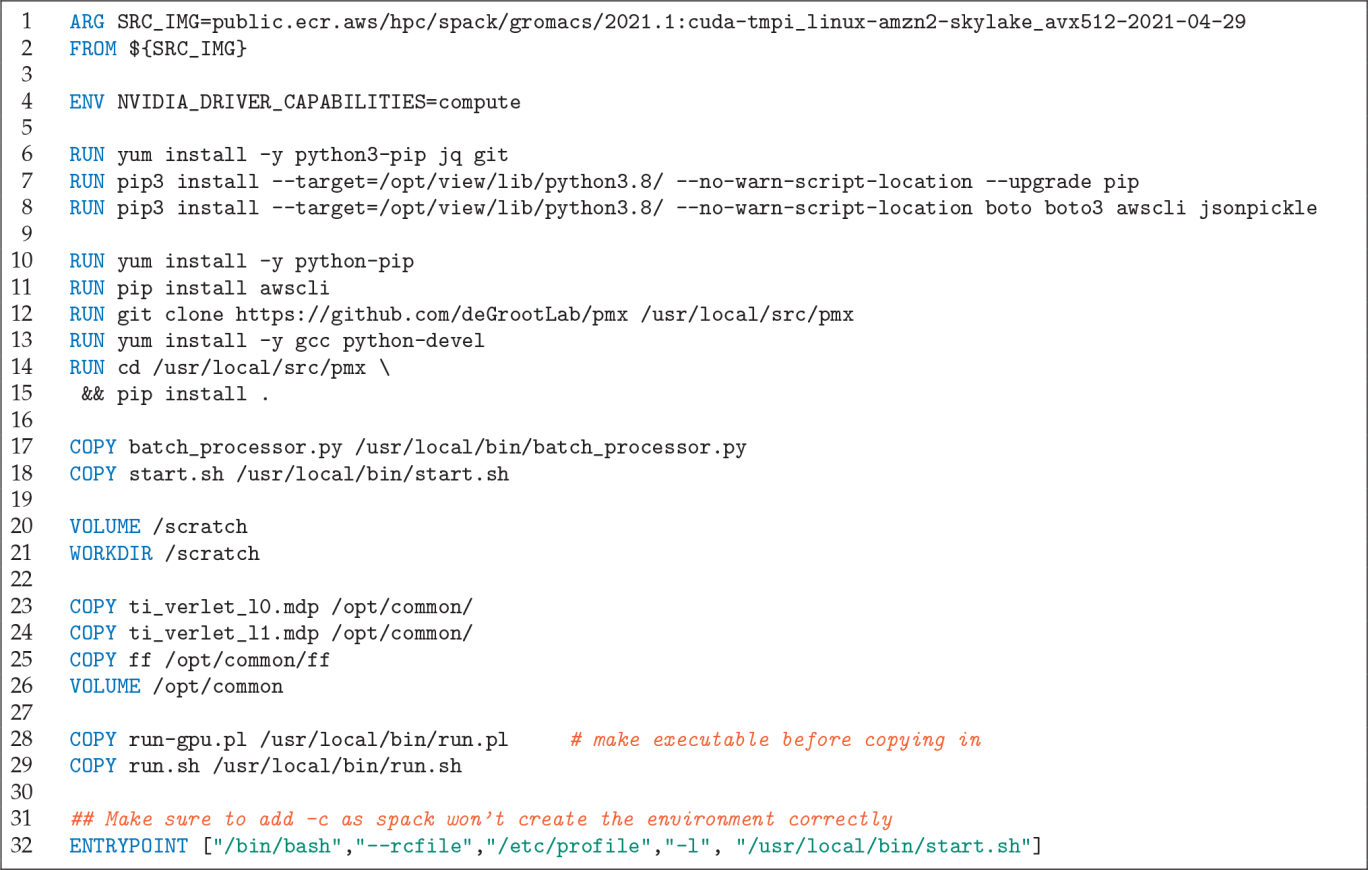
Example of a Docker file for a GPU image. From the Docker files multiple Docker container images are compiled (one for each architecture) that are loaded from the Amazon ECR by the instances.

**Figure 3:**
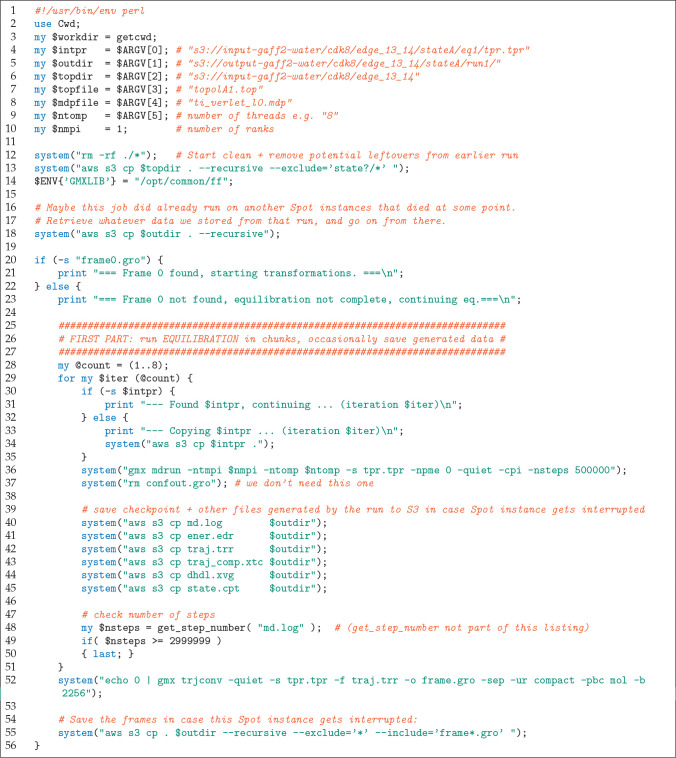
Perl script used to launch each of the 19 872 jobs (first part).

**Figure 4:**
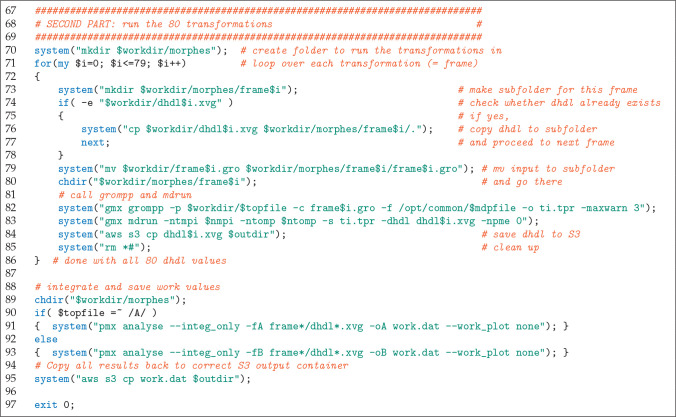
Perl script used to launch each of the 19 872 jobs (cont’d).

For the binding affinity ensemble, we used Spot instances because they are much cheaper than on-demand. The downside of a Spot instance is that it can be terminated at any time, which can happen if the pool of free Spot instances shrinks over time and more on-demand capacity is requested in a region. To minimize instance termination, we requested a number of instances in each region proportional to the Spot pool size of that region. We introduced additional flexibility by requesting instances with all possible vCPU counts and fitting several jobs on them. A single 96 vCPU c5.24xl instance could then, for example, end up running one 48 vCPU job plus six 8 vCPU jobs at the same time. To better understand the whole setup, let us look at the encircled digits (red) in Figure 1 and follow the lifetime of one of the 19 872 jobs from the binding affinity ensemble. ① We submit an example job from a Cloud9 terminal to the HyperBatch entry point. The job definition file specifies how many vCPUs to allocate, whether to request a GPU, and which subfolders to use in the S3 input and output buckets for job I/O. HyperBatch distributes jobs across regions according to region weights reflecting the compute capacity of the regions, *e.g.*, using the weights (6, 6, 3, 1, 1, 4) for the regions (us-east-1,us-east-2,us-west-2,ap-southeast-1,ap-northeast-2,eu-west-1) for GPU jobs. Our example job gets distributed to eu-west-1 (blue, ②), where it is relayed to a Batch instance ③ with sufficient free resources (vCPUs, GPUs). The instance loads the correct Docker image from AWS Elastic Container Registry (ECR) with preinstalled software for the current architecture ④, *e.g.*, pmx and the GROMACS executable with the SIMD level matching the CPU (see Figure 2 for the definition of the Docker file). The actual simulations are handled by the Perl script shown in Figures 3 and 4. This script is designed to deal with sudden interrupts that are possible with Spot instances. Accordingly, output data and checkpoints are saved in regular intervals to S3 storage. To start a simulation, step ⑤ loads the input files from S3 (line 13 in Figure 3). Step ⑥ loads potentially existing output data from S3 (line 18 in the listing); this is the case when the job was running earlier already but was interrupted before it finished. Depending on whether prior output data is present, the job is either continued or started from scratch. Generally, the MD job consists of two parts: (i) the production of an equilibrium trajectory (lines 25–56 in the listing) and (ii) the 80 individual transformations (lines 67–86). Part i is executed in eight chunks (lines 28 and 29) so that upon instance termination only a small part needs to be recomputed, as ⑦ each chunk’s data is transferred to S3. If an instance terminates during one of the 80 transformations, the job is continued from the start of that transformation, as a completed transformation ⑦ is immediately saved to S3. At last, pmx integrates and saves the work values that are later used for free energy estimation (lines 88–95). Instance termination ⑧ at any time will trigger a Lambda function that resubmits the job again to HyperBatch. The current state of each job can be checked in a DynamoDB table (Figure 1, right). Additional configuration using Amazon Elasticsearch allows globally monitoring the whole simulation ensemble in a Kibana^68^ dashboard that shows the total number of running instances, the instance types by region, and more.

## 4 Results and Discussion

We present our results in four parts: (i) performance, scaling, and cost efficiency in terms of performance-to-price (P/P) ratios for the standard MD input systems such as MEM and RIB on CPU and GPU instances; (ii) a cost comparison of cloud computing versus buying and operating an own cluster; (iii) as a prerequisite for the binding affinity study, the results of the free energy benchmarks (SHP-2, c-Met, and HIF-2α) on various instance types, including the resulting performance-to-price ratios; (iv) the performance and the costs of the binding affinity studies on global cloud resources.

### 4.1 Which Instances Are Optimal for GROMACS?

Tables 5–9 show the benchmark results for various instance types. For CPU instances, Table 5 lists MEM and RIB performances in gray and blue colors, and the resulting P/P ratios from greens over yellows to reds, corresponding to high, medium, and low cost efficiency. Table 6 shows the same for instances with up to 8 GPUs. As the mapping of colors to values depends on the smallest and largest observed values, it differs between MEM and RIB, but is the same across all tables. As a result, greens will always refer to good choices in terms of P/P ratio. For several of the instances, various ranks × threads decompositions are listed; “PME ranks” indicates if and how many MPI ranks were set aside for PME.

**Table 5:**
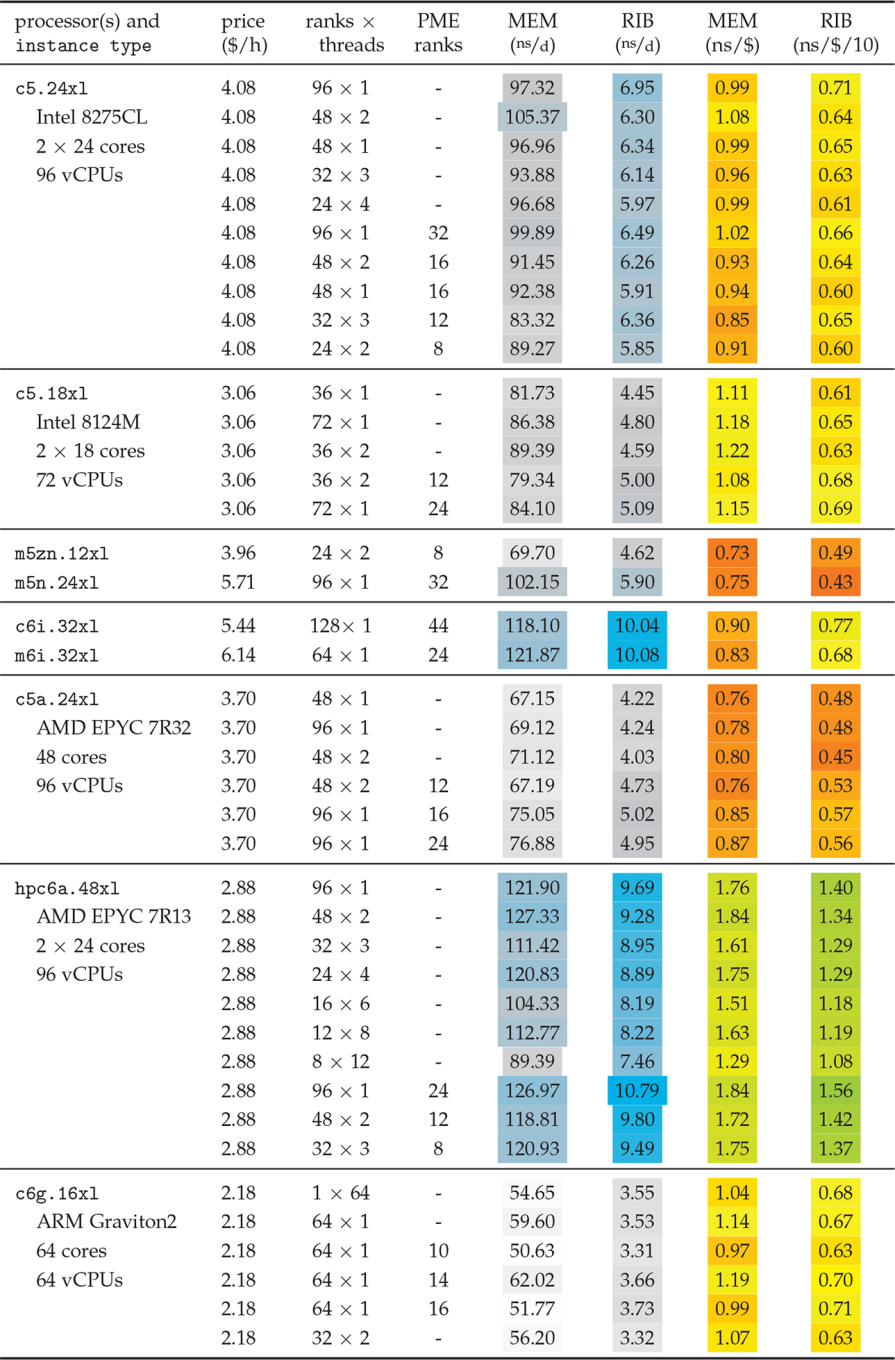
GROMACS 2020 Performance on Selected CPU Instances^a^

a ns/d values list MEM and RIB performances, and (ns/$) columns show performance to price. Values are color-coded for a quick visual orientation: Grays for low performances, blue towards higher values. For the performance-to-price ratios, reds indicate sub-average ratios, yellows average, and greens above-average ratios.

#### 4.1.1 Performance on Individual Instances with CPUs

The highest performances were measured on hpc6a.48xl, c6i.32xl, and m6i.32xl instances, which is expected as with 96–128 vCPUs they offer the largest number of cores (see also Table 1). Performance-wise, they are followed by 96 vCPU c5.24xl and m5n.24xl instances. In terms of cost-efficiency, hpc6a instances are the clear favorites among the CPU instances.

#### 4.1.2 Performance on Single Instances with GPUs

From the GPU instances (Table 6), the g5 with 8–32 vCPUs reach or even surpass the performance of the hpc6a.48xl CPU instance, albeit with a significantly (1.25–2.4×) better cost-efficiency. In fact, the single-GPU g4dn’s with 4–16 vCPUs and g5’s with 4–32 vCPUs exhibit the best cost-efficiency of all instances for the MEM and RIB benchmarks. Perhaps unsurprisingly, the highest single-instance performances of this whole study have been measured on instances with eight GPUs. With the exception of the (comparatively cheap) quadruple-GPU g4dn.12xl instances, however, the P/P ratio plunges when distributing a simulation across multiple GPUs on an instance. In those cases, GROMACS uses both domain decomposition *via* MPI ranks as well as OpenMP parallelization, with added overheads of both approaches. Additionally, as the PME long-range contribution can not (yet) be distributed to multiple GPUs, it is offloaded to a single GPU, while the other GPUs share the remaining calculations of the nonbonded interactions. All imbalances in computational load between the GPUs or between the CPU and GPU part translate into a loss in efficiency and thus in a reduced cost-efficiency. For single-GPU simulations, GROMACS has a performance sweet spot. Here, domain decomposition is usually not needed nor invoked, and all nonbonded interactions including PME can be offloaded to a single GPU, leading to considerably less imbalance than in the multi-GPU scenario. To use instances with *N* GPUs more efficiently, one can run *N* simulations simultaneously on them *via* GROMACS’ built-in -*multidir* functionality, thus essentially gaining the efficiency of the single-GPU case. This is demonstrated in Table 6 for the p4d.24xl and the g4dn.12xl instances. The p4d.24xl line in the table shows the results for parallelizing a single simulation across the whole instance, whereas p4d.24xl/8 shows what happens when eight simulations run concurrently. Here, the produced amount of trajectory and thus also the cost-efficiency, is about four times as high. For the g4dn.12xl/4 vs g4dn.12xl instance, running four concurrent simulations instead of one simulation translates into about a factor of 2 higher cost-efficiency.

**Table 6:**
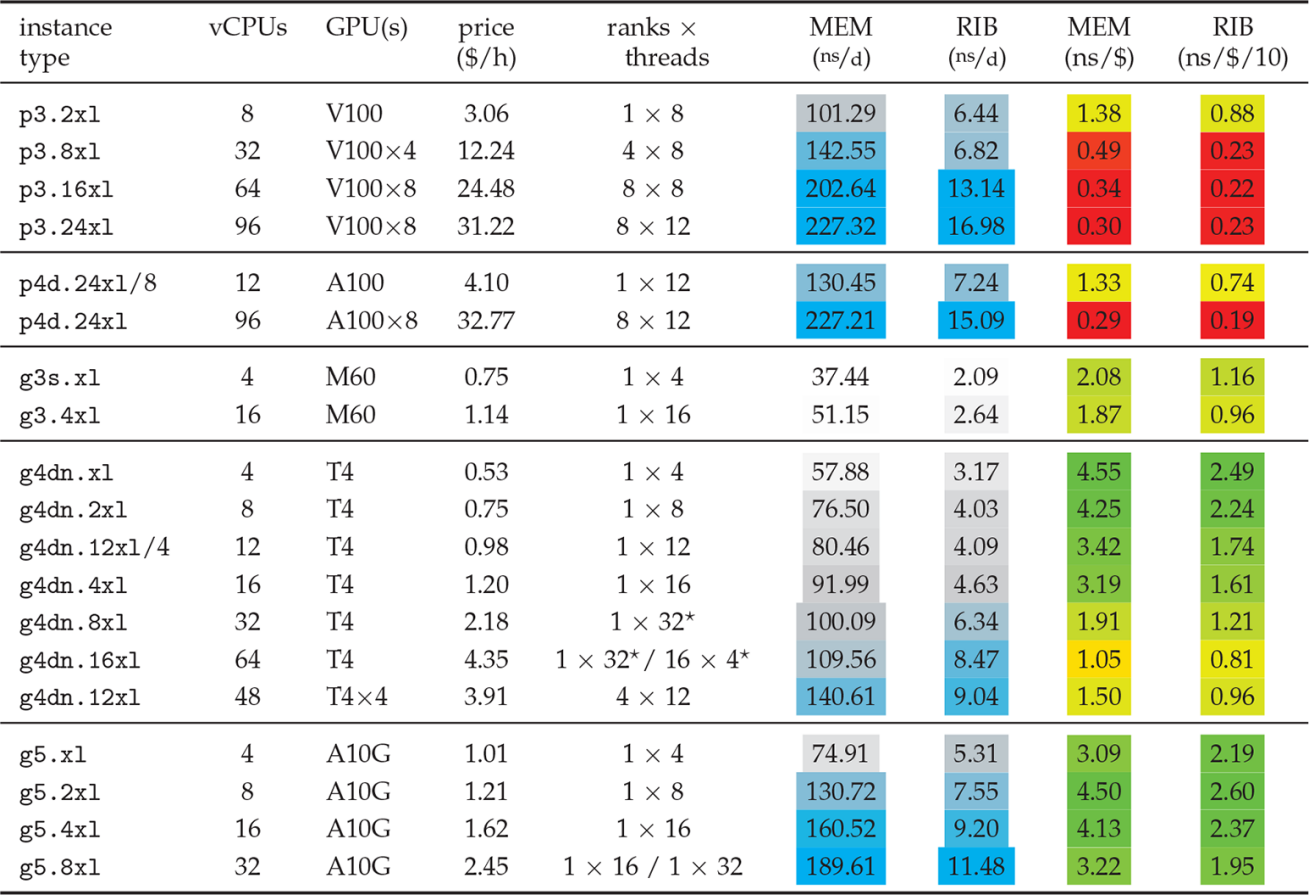
GROMACS 2020 Performance on Individual Instances with GPUs^a^

a As in Table 5, but on instances with up to eight GPUs. PME long-range interactions were offloaded to a GPU in all cases, except *, where they were evaluated on the CPU.

#### 4.1.3 Scaling Across Multiple Instances

For selected instance types, we also determined how much performance can be gained on multiple instances. For this we have selected instance types that (i) exhibit above average P/P ratios for the single-instance benchmarks and (ii) have a network speed of at least 50 Gigabit/s.

Tables 7, 8, and 9 summarize the results for scaling across 1–32 CPU and GPU instances. For the 81 k atom MEM system, the maximal performance is reached on 4 hpc6a instances, however at a parallel efficiency of less than 40%, whereas for the g4dn’s, the highest performance is recorded on individual instances. In contrast, the large RIB system shows a decent scaling behavior. On hpc6a, the single-instance performance of 10.8 ns/d can be increased to about 60 ns/d at a parallel efficiency of 69% on eight instances. On 32 instances, with 115 ns/d, the single-instance performance is increased 11-fold. Whereas the RIB system continues to scale beyond 8 hpc6a instances, the g4dn’s never reach 30 ns/d. The difference in scaling efficiency between CPU and GPU instances is mainly determined by the network speed for the internode communication. As the hpc6a instances have a much better interconnect than g4dn (see Table 1), the scaling is more efficient for the CPU nodes. The hpc6a instances, however, never reach the scaling performance of an on-premises dedicated HPC cluster. There, as shown in Figure 7 of ref (43), the same benchmark systems exhibit peak performances of 303 ns/d for MEM and 204 ns/d for RIB.

**Table 7:** Scaling Across Multiple CPU Instances^a^

instances	total vCPUs	ranks × threads	PME ranks	MEM (ns/d)	*E*_MEM_	RIB (ns/d)	*E*_RIB_
1	96	48 × 2/96 × 1	0/24	127.5	1.00	10.81	1.00
2	192	48 × 4/192 × 1	12/48	158.8	0.62	20.35	0.94
4	384	48 × 8/384 × 1	12/96	201.1	0.39	37.63	0.87
8	768	384 × 2	96	182.9	0.18	59.93	0.69
16	1536	128 × 12/384 × 4	32/96	151.9	0.07	87.21	0.50
32	3072	384 × 8/768 × 4	96/192	144.3	0.04	115.49	0.33

a GROMACS 2020 performances for MEM and RIB over multiple hpc6a instances. The third column lists the optimal decomposition into MPI ranks and OpenMP threads, and the fourth column lists the optimal number of separate PME ranks; the left entry is for MEM, the right entry for RIB if they differ.

**Table 8:** Scaling Across Multiple GPU Instances^a^

instances	total cores	ranks × threads	MEM (ns/d)	*E*_MEM_	RIB (ns/d)	*E*_RIB_
1	16	1 × 16/16 × 1	95.3	95.3	5.15	1.00
2	32	4 × 8	65.0	65.0	8.49	0.82
4	64	8 × 8/32 × 2	73.1	73.1	15.80	0.77
8	128	32 × 4/64 × 2	63.7	63.7	21.25	0.52
16	256	32 × 8			25.86	0.31
32	512	32 × 16			22.78	0.14

a As in Table 7, but for g4dn.8xl instances with hyperthreading off.

**Table 9:** Scaling Across Multiple GPU Instances^a^

instances	total cores	ranks × threads	MEM (ns/d)	*E*_MEM_	RIB (ns/d)	*E*_RIB_
1	32	1 × 32/8 × 4	98.1	1.00	7.48	1.00
2	64	8 × 8/32 × 2	76.0	0.39	13.27	0.89
4	128	8 × 16/32 × 4	73.2	0.19	19.50	0.65
8	256	32 × 8			24.39	0.41
16	512	64 × 8			28.38	0.24
32	1024	32 × 32			21.47	0.09

a As in Table 7, but for g4dn.16xl instances with hyperthreading off.

**Figure 5:**
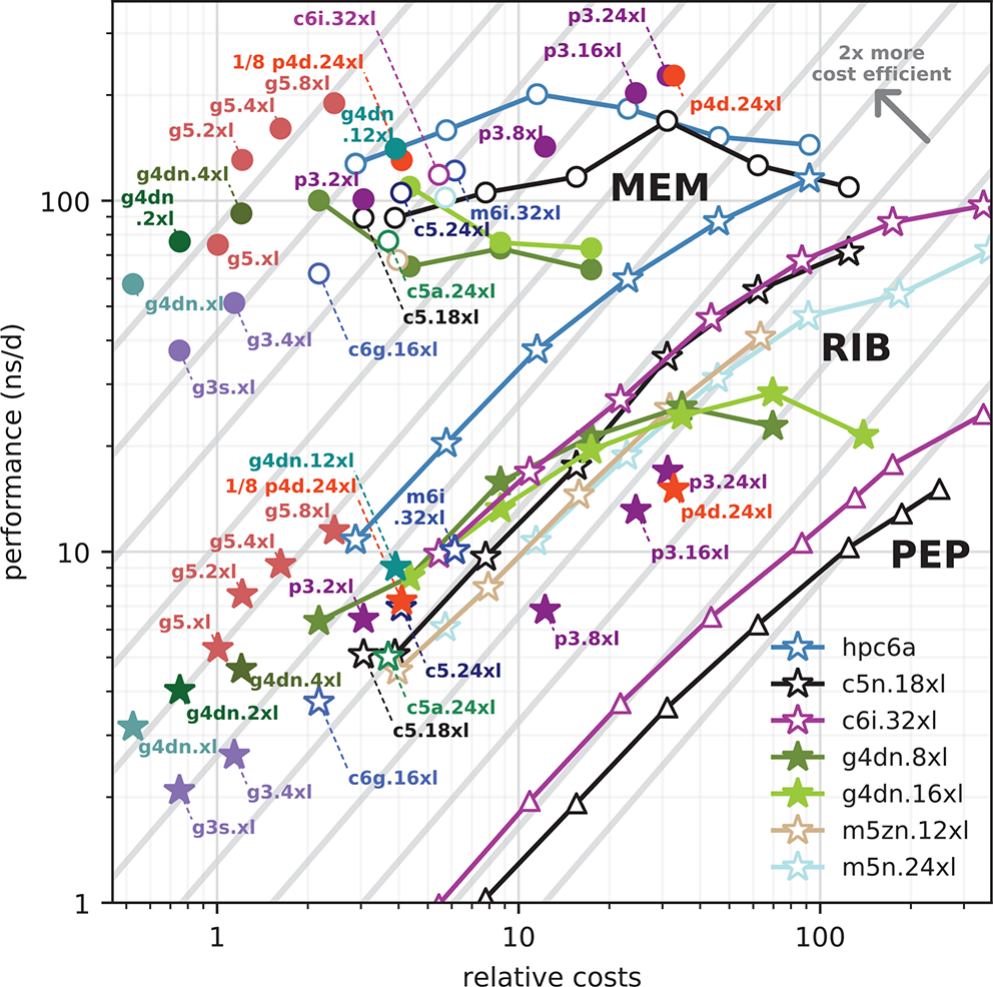
Performance, costs, and cost-efficiency for GROMACS simulations on various AWS instance types. GROMACS 2020 performance as a function of the on-demand instance costs ($/h) for the MEM (circles), RIB (stars), and PEP (triangles) benchmark on CPU (open symbols) and GPU instances (filled symbols). Separate symbols indicate single-instances; connected symbols show the parallel scaling across multiple instances.

Figure 5 summarizes all benchmark results and interrelates them to uncover which instances are optimal in terms of both performance and cost-efficiency. The symbols show benchmark performances (at optimal parallelization settings) on various instances as a function of the on-demand hourly instance costs. The inclined gray lines are isolines of equal P/P ratio with the most cost-efficient configurations toward the upper left. Moving from one isoline to the neighboring one toward the top left improves the P/P ratio by a factor of 2. Symbols connected by a line denote the strong scaling behavior across multiple identical instances, with a single instance at the left end of the curve, followed by 2, 4, 8, and so on, instances. A scaling curve that runs parallel to the cost-efficiency isolines would indicate optimal scaling, *i.e.*, a parallel efficiency of *E* = 1. Figure 5 allows a series of observations. (i) In terms of cost-efficiency, the optimal instances for GROMACS are the single-GPU g4dn’s with 4, 8, and 16 vCPUs (green symbols toward the left) and g5’s (brown symbols) whose P/P ratio is at least a factor of 2 higher than most of the other instance types. (ii) Perhaps unsurprisingly, the highest MEM and RIB performances on individual instances are reached with p3 and p4d instances hosting eight GPUs connected *via* PCI Express (red and purple symbols). (iii) For larger systems (RIB and PEP), the highest absolute performances are reached by scaling across multiple c6i.32xl or hpc6a.48xl instances, with the hpc6a’s showing by far the best cost-efficiency. (iv) The performance of small systems like MEM cannot be significantly improved by scaling across many instances. (v) Choosing one of the many possible instances for an MD project essentially boils down to pinning down a point along the connecting line between best cost-efficiency and highest performance, trading off HTC and HPC computing. Let us follow this special line for the example of the RIB benchmark. It starts at optimal cost-efficiency with the single-GPU g5.2xl instances (left, brown stars). For higher performances, one would pick g5.4xl and then g5.8xl instances, however at the cost of losing 10%–25% in P/P ratio. For higher performances (again, at reduced cost-efficiency), the scientist would then continue with scaling over hpc6a instances (blue) which exhibit the best P/P ratios toward growing performances. There is generally no reason to pick instances within the area below the described line as here one simply gets lower GROMACS performance for the same price. For example, for the price of a g3.4xl instance (violet, bottom left), one could instead choose a g5.xl or g5.2xl that exhibits two times the RIB performance.

### 4.2 Cost Comparison: Cloud vs On-Premises Cluster

Whether it is more cost-efficient to run simulations on a cloud-based cluster depends of course almost completely on the specific use case, *i.e.*, how big the cluster will be, what software will run on it, and whether there are enough jobs at all times to keep the cluster busy as opposed to bursts of compute demand with idle time in between. Therefore, no generalizable results or guidance can be provided here. We do think, however, that rough estimates of respective costs and comparison to a typical local compute cluster at a research institution will provide useful information and guidelines in particular for new groups in the field who need to set up computer resources. To this aim, we will estimate and compare the total costs of producing one microsecond of trajectory for the RIB benchmark with GROMACS.

**Figure 6:**
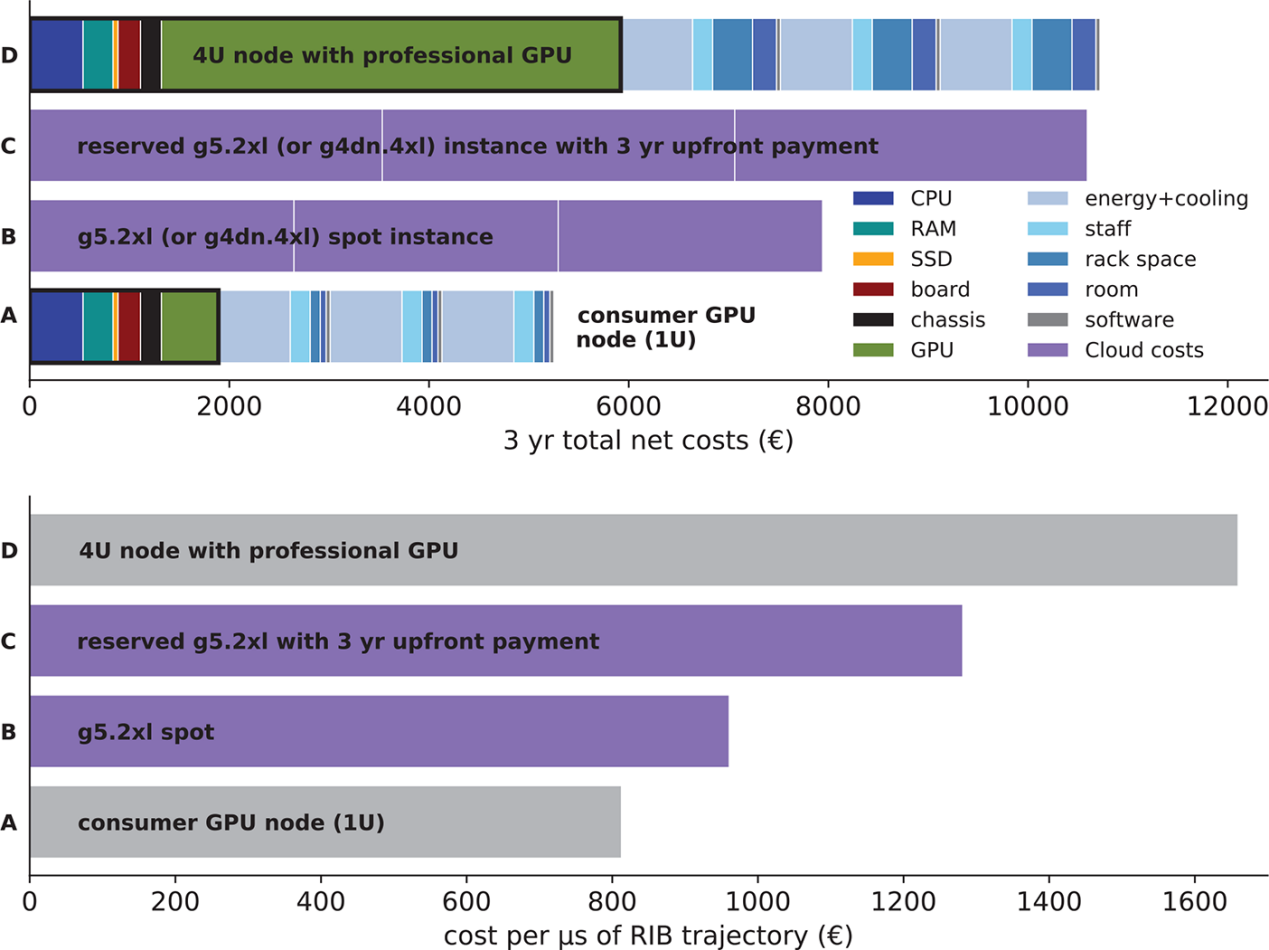
Costs and cost-efficiency of a compute node in an owned cluster compared to a cloud instance with similar GROMACS performance over 3 years. Top panel: Violet bars show costs of AWS g5.2xl instances (producing 7.55 ns/d of RIB trajectory), which offer one of the highest performance-to-price ratios for GROMACS (compare Figure 5), in individual blocks of one year. Bar A shows the fixed costs for buying a consumer GPU node tailored to GROMACS within the thick black line (broken down into individual hardware components) plus the yearly recurring costs (mainly energy) for three years. This node (E5-2630v4 CPU plus RTX 2080 GPU) produces 5.9 ns/d of RIB trajectory.^41^ Bar B shows the average costs using an AWS Spot instance. Bar C shows the costs when reserving the AWS instance and paying up front. Bar D is the same as bar A, but using a 4 U node with a professional GPU (*e.g.*, Quadro P6000). Bars A–D in the lower panel show the resulting RIB trajectory costs for the nodes shown in the top panel, for a service life of three years.

The hardware for an own cluster can be aggressively tuned toward cost-efficiency for simulations with GROMACS. Combining inexpensive processors with consumer GPUs yields the best performance-to-price ratios.^41^ For instance, 1 U (rack unit) nodes with an Intel E5-2630v4 processor plus an NVIDIA GeForce RTX 2080 GPU were offered for under 2000 € net at the time, including three years of warranty. Investment costs for the racks, cooling system, and infrastructure needed to operate the cluster are estimated to about 500 € per U of rack space over the lifetime of the racks. For a lifetime of 5 years, that adds 100 € per U per year. For technical staff to operate, repair, and look after a 500 node cluster, we assume 100 000 € per year, which adds 200 € to the operating costs for each node per year. A suitable room (60–100 m^2^ for about 500 U of hardware with appropriate infrastructure and the possibility to install heavy apparatus) adds about 30 000 € to the yearly costs (60 € per node), depending on the location. For cluster management software we assume 40 € per node per year. Bar A of the top panel of Figure 6 shows a breakdown of the total costs for our optimized consumer GPU node. Bar D illustrates how the costs grow when using the same hardware as in bar A, but now with a professional GPU (*e.g.*, an NVIDIA Quadro P6000) instead of a consumer GPU (which leads to considerably higher fixed costs) and in a larger chassis that takes 4 U rack space (which lead to significantly increased recurring costs for room and rack space). Thus, densely packed hardware helps to reduce costs. The lower panel of Figure 6 shows the resulting costs per microsecond of RIB trajectory for the nodes from the upper panel. g5.2xl instances offer both a high absolute performance as well as a good performance-to-price ratio for GROMACS (Figure 5), which would therefore be a good pick for production runs. However, on-demand g5.2xl instances would yield RIB trajectory costs as high as 3200 €/μs. To reduce costs, one would reserve an instance for one or three years, and for maximal savings one can pay up front (bars C). g5.2xl Spot instances are nearly as cost-efficient as consumer-GPU nodes tailored toward GROMACS (bars A and B in the lower panel). They are more cost-efficient than buying a node with a professional GPU (bar D). In summary, with careful selection of cloud resources and payment options, there is not much difference in cost today compared to on-premises computing.

### 4.3 GROMACS Performance for Free Energy Calculations

Turning on FE perturbations reduces the GROMACS performance, because an additional PME grid is evaluated, and because interactions involving perturbed atoms run through kernels that are not as optimized as the standard kernels. How much the performance differs with and without FE depends on how big the fraction of perturbed atoms is and on the parameters chosen for FE. For those reasons we cannot use the MEM and RIB benchmarks to predict the performance of the systems used in our high-throughput ligand screening study. Instead, we carry out new benchmarks for four representative FE systems (Table 2) chosen from the whole binding affinity ensemble (Table 3). The performances for these systems, which are a small ligand-in-water system (from the c-Met data set) plus three protein–ligand complexes of different size (HIF-2α, c-Met, and SHP-2) are shown in Table 10 for CPU instances for various decompositions into MPI ranks and OpenMP threads. For convenient navigation and the latest updates of the benchmark data we also provide access to this information *via* web interface: http://pmx.mpibpc.mpg.de/aws.pl. The table shows the general trend of small instances exhibiting higher P/P ratios, but there are no pronounced differences between the architectures. The highest performances are observed on the 96 vCPU Intel instances.

**Table 10:**
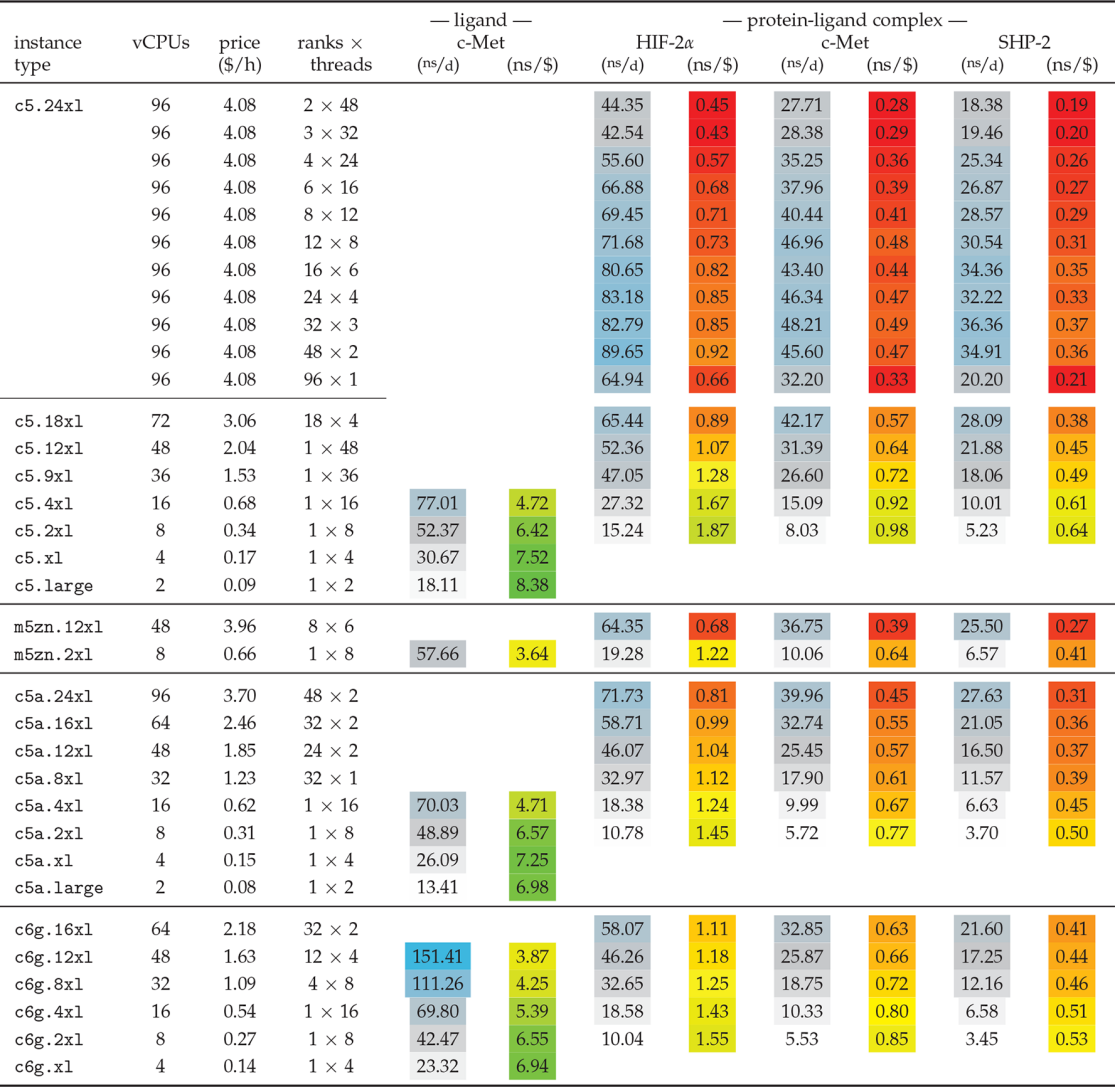
Free Energy Benchmarks on CPU Instances^a^

a Performance (ns/d) and performance-to-price ratios (ns/$) for GROMACS 2020 on various Intel (c5 and m5zn), AMD (c5a), and ARM (c6g) CPU instances. Color coding as in Table 5.

**Figure 7:**
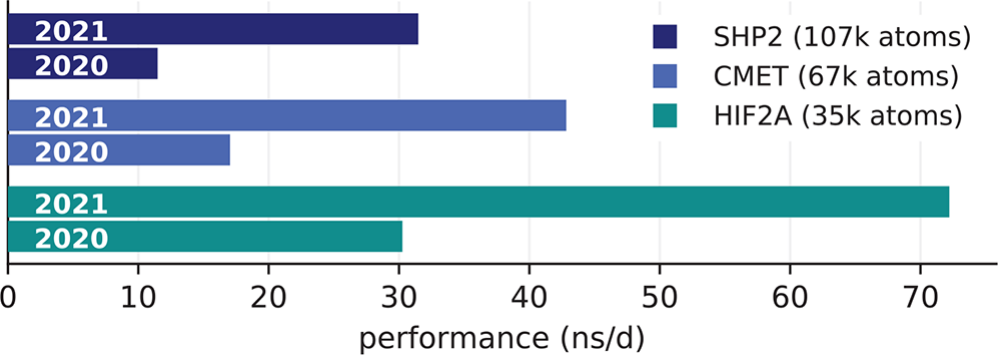
Performance improvements of GROMACS 2021 for free energy calculations on GPUs. For three different MD systems (colors) with free energy perturbation turned on, the bars compare GROMACS 2021 and 2020 performances on a p3.2xl instance.

Up to version 2020, with perturbed charges it was not possible to offload the PME grid calculations to the GPU. This has changed from version 2021 on, leading to considerably enhanced performance (more than a factor of 2) on GPU instances in our cases (Figure 7). Therefore, we used GROMACS 2021 for all binding affinity simulations. The benchmark results for the four representative FE systems on GPU instances are assembled in Table 11.

**Table 11:**
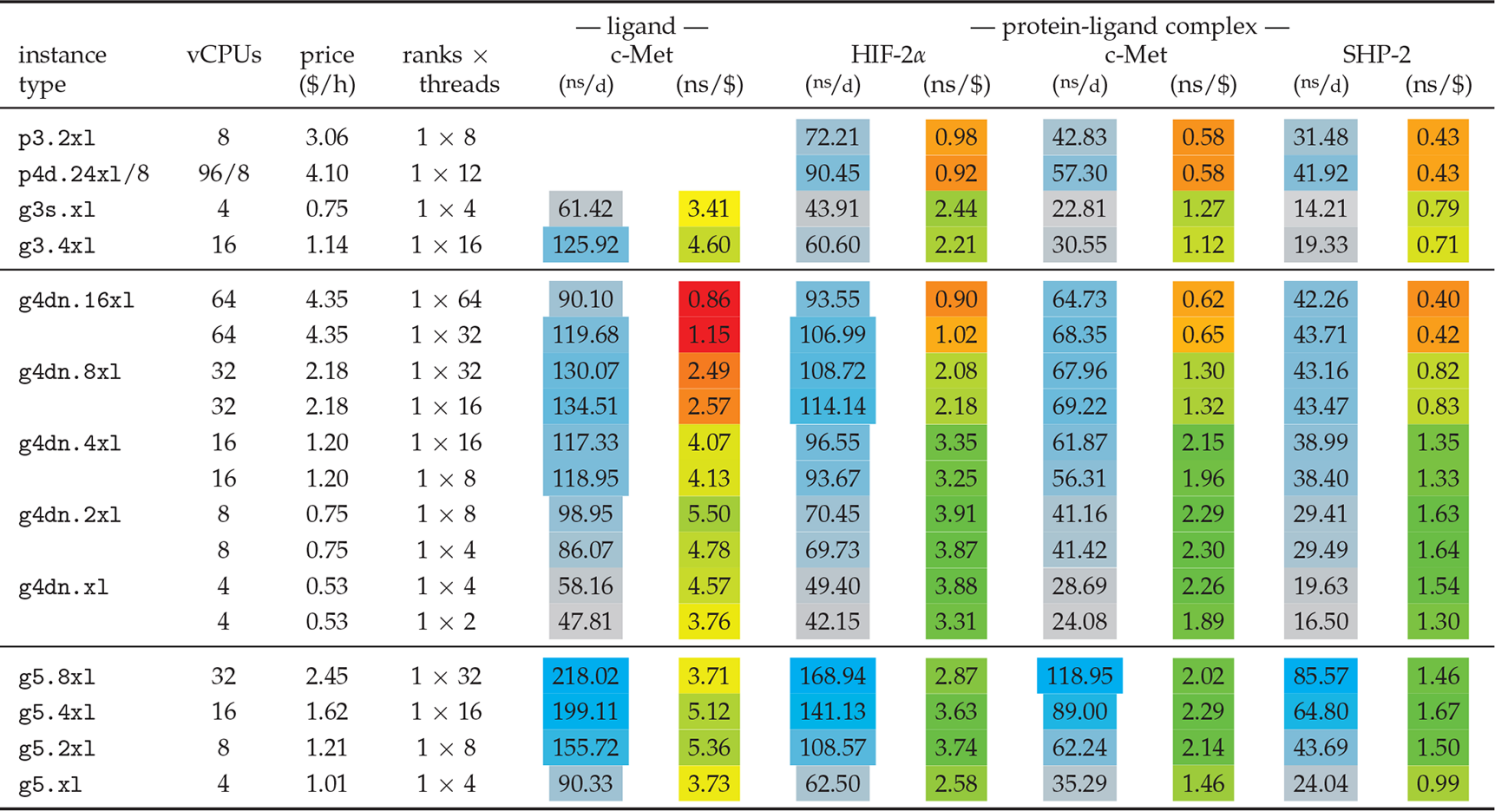
Free Energy Benchmarks on GPU Instances^a^

a As in Table 10, but now for GROMACS 2021 using one GPU per simulation. The single-GPU performance on p4d.24xl was derived by running 8 identical benchmarks, each using one GPU and 1/8th of the hardware threads, in a multi-simulation.

Whereas the performances of the 32 and 64 vCPU g4dn instances are comparable to or higher than that of the best performing CPU instances (*i.e.*, c6g.12xl for the ligand in water and c5.24xl for the protein–ligand complexes), the smaller g4dn instances with ≤16 vCPUs still offer high performance but at exceptionally high P/P ratios: about two times higher than on CPU instances. On the instances with ≥32 vCPUs it is beneficial for performance to just use half the number of vCPUs for OpenMP threads, as the reduction of values over too many threads can deteriorate performance otherwise. The recently introduced g5 instances even surpass the g4dn performance at a similarly good price–performance ratio. Regarding cost-efficiency, any of the c5, c5a, or c6g instances with ≤8 vCPUs has a high P/P ratio for the small ligand-in-water systems, whereas single-GPU g4dn instances with ≤16 vCPUs or g5’s with 8–32 vCPUs are undefeated for the larger protein–ligand systems.

#### 4.3.1 Costs and Time-to-Solution per FE Calculation

**Figure 8:**
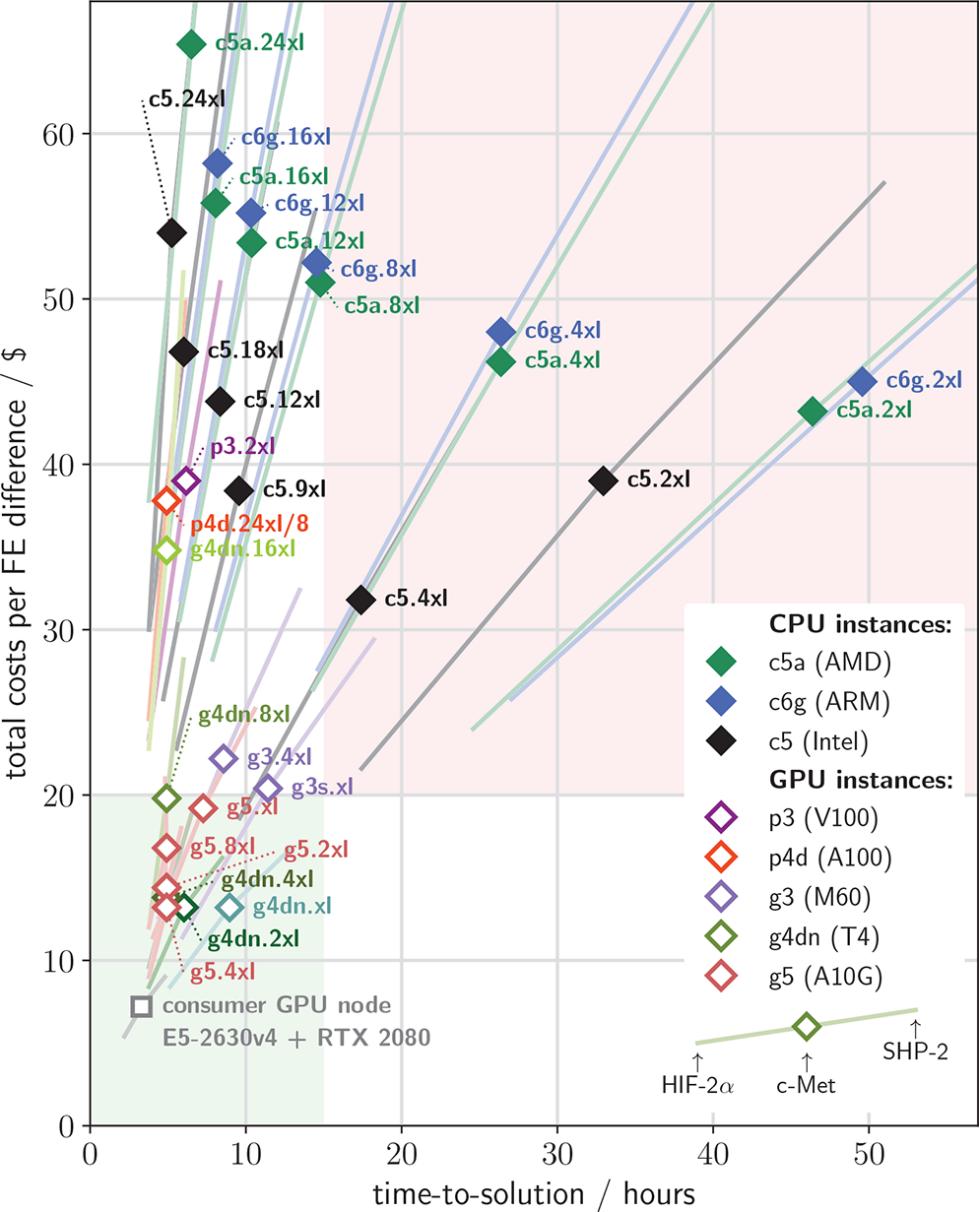
Costs and time needed to compute one FE difference. Diamonds show the costs to compute one FE difference (using Spot pricing) versus the time-to-solution for various instance types (colors) for the c-Met system. In addition to c-Met, HIF-2α is shown at the lower left end of each colored line, and SHP-2 at the upper right end. The gray square shows costs and timings for a consumer GPU node specifically tuned for GROMACS simulations, as discussed in section 4.2 and shown in Figure 6A.

The numbers in Tables 10 and 11 are for the equilibration phase of the FE calculation (see section 3.3.2). We do not list the benchmark results of the transformation phase separately, but included them in the estimate of the total cost of computing one FE difference, as described in Methods. Figure 8 shows the time-to-solution and the costs per FE difference that result when using different Spot instance types. The figure does not include hpc6a instances because currently hpc6a is available only on-demand. With three replicas and two directions, the total costs for one FE difference is 6× the time needed for the protein–ligand part, plus the (small) costs of the ligand in water.

Spot instance costs are just about a third of the on-demand costs (not shown), although Spot prices vary slightly among the regions and change over time. We therefore used Spot instances for our binding affinity studies, even though these may be terminated at any time should there be demand for that instance type in the given region. As can be seen in Figure 8, on CPU instances the time-to-solution generally shrinks with the number of vCPUs (as expected) while the costs grow. Using g5.2xl, g5.4xl, g4dn.xl, g4dn.2xl, or g4dn.4xl GPU instances, any FE calculation is completed within 15 h for less than $20 for all systems (green quadrant). Other single-GPU instances like g4dn.8xl, g5.8xl, and g5.xl are somewhat less cost-efficient but still better than the remaining instance types. The white quadrant on top of the green quadrant accommodates multiple instance types on which an FE value can be computed in less that 15 h, albeit at a markedly higher cost than on g4dn instances.

### 4.4 High-Throughput Ligand Screening in the Global Cloud

#### 4.4.1 Study 1: Focus on Time-to-Solution

Our first screening study consisted of 19 872 Batch jobs to compute 1 656 free energy differences (200 μs of trajectory in total) for the ensemble shown in Table 3. With this study we evaluate the suitability of cloud computing for large-scale computational drug design scans that have been traditionally performed on on-premises clusters where such a scan would typically take several weeks to complete.

**Figure 9:**
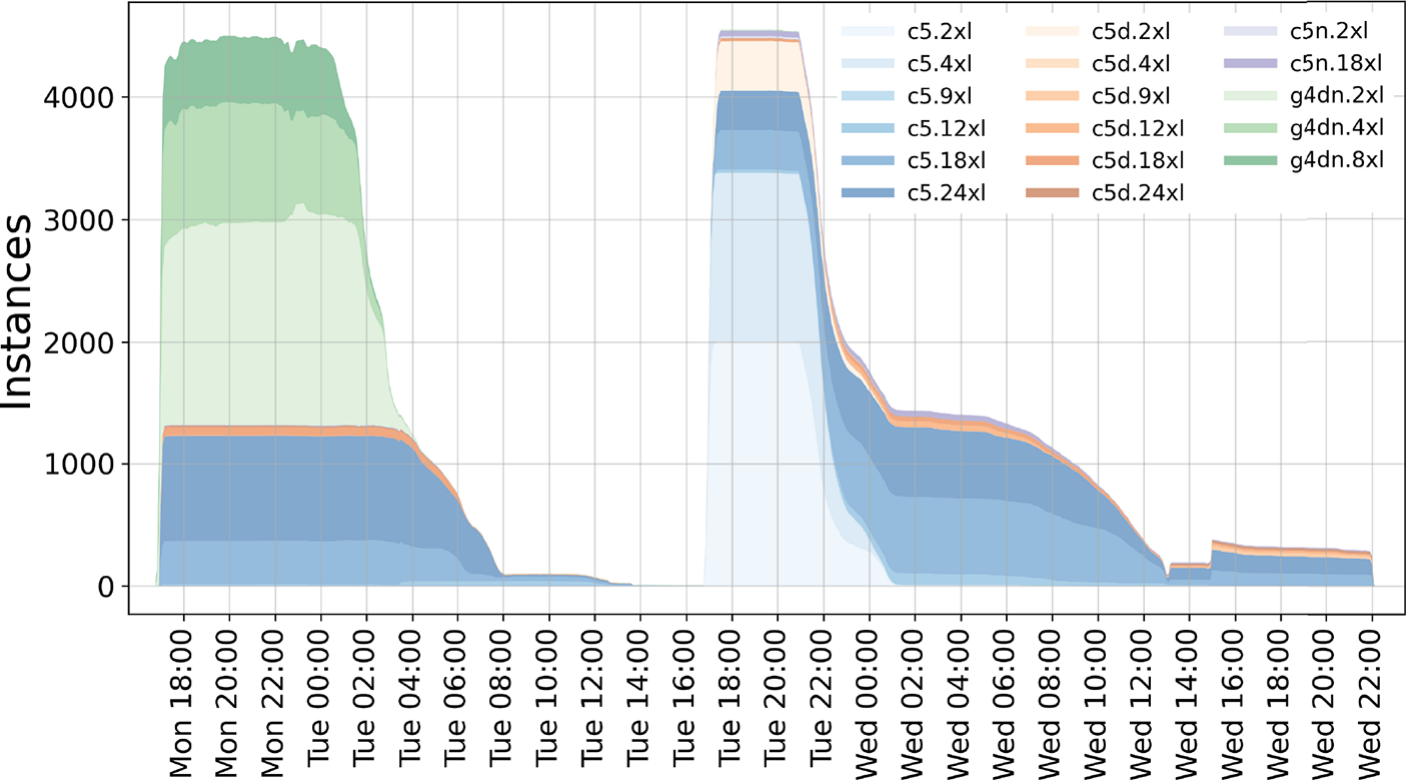
Usage of global compute resources for the first ligand screening study aimed to optimize time-to-solution. Colors show the various instances that were in use globally during the 3 days of the ensemble run.

As we aimed to minimize the time-to-solution, from all available instance types we selected only instances that would need no more than nine hours for any job. The g4dn.2xl, g4dn.4xl, and g4dn.8xl meet that criterion at the lowest costs. (At the time of our screening studies the g5 GPU instances were not yet available.) However, relying on just three instance types is risky if one wants to minimize time-to-solution. g4dn instances are not very abundant in the AWS cloud, and if they happen to be in high demand at the time of our screening study, we might not get many of them. Therefore, we added other instance types that meet our nine hour criterion but that are almost always available: c5.24xl and c5.18xl as well as the similar c5d.24xl and c5d.18xl. We ran the small systems of ligand in water on eight c5 vCPUs, where they would complete in about five hours at a price of less than $2 and high cost-efficiency (see c-Met ligand column in Table 10). To draw from a larger pool of instances we allowed for c5 instances of various size and just requested that they offer at least eight vCPUs (see also Figure 9). Larger instances accept multiple jobs until they do not have enough free vCPUs left.

**Figure 10:**
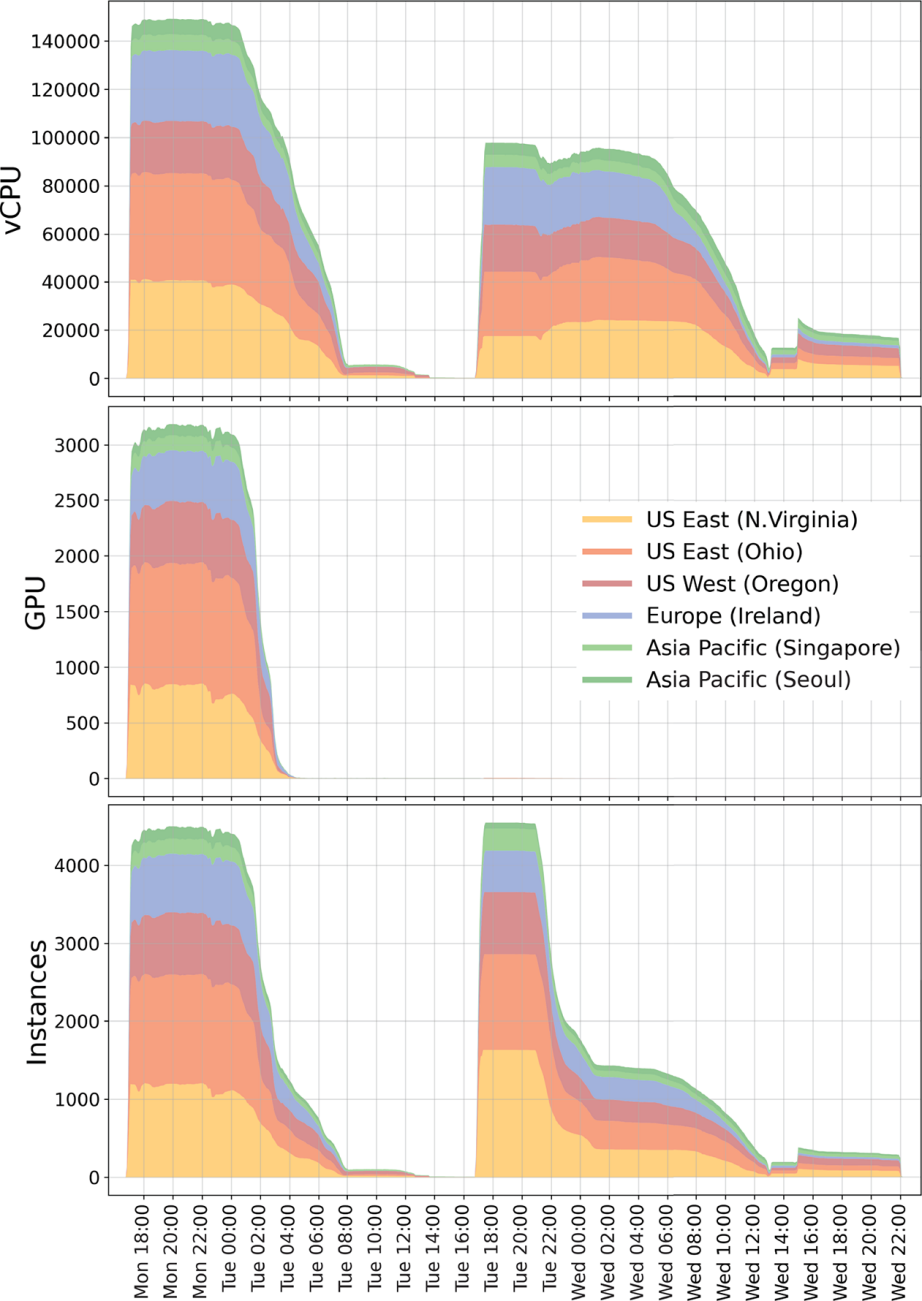
Usage of global compute resources for the first ligand screening study aimed to optimize time-to-solution. Compute resources (split into regions) allocated for the ensemble run over time. Top, vCPUs; middle, GPU instances; bottom, number of instances.

We submitted the first 9936 jobs (the large protein systems) in a first wave on a Monday at around 5 p.m., and the second 9936 jobs (the small ligand systems) were submitted in a second wave 24 h later. Figure 9 shows the number of instances that were in use during our first screening study color-coded by instance type. Figure 10 provides further details of the run. As can be seen from the top and middle panels, we acquired about 140 000 vCPUs within the first 30 min and about 3000 GPUs within the first two hours of the run, distributed globally over six regions.

Each wave finished in about 1 day, and we speculate that also the whole 19 872 jobs would have finished within 24 h if submitted simultaneously. As GPU instance availability is essentially independent of the CPU instance availability, the GPU jobs from the first wave (greens in Figure 9) can completely overlap with the CPU jobs of the second wave. At the same time, after the peak of the second wave (Tue 17 h–23 h), there should be more than enough c5 Spot capacity to accommodate the CPU jobs of the first wave.

Unfortunately, there was a glitch in the initial version of our setup that prevented finished instances to terminate properly. For that reason, the actual costs of the first run summed up to $56 per FE difference, although, when counting productive time only, they reduce to $40 per FE difference. This is in the expected range (see Figure 8), given the mix of instances that were in use. The overall costs almost entirely resulted from the hourly charges of the EC2 compute instances, whereas data transfer to and from the S3 buckets accounted for less than 0.5% of the whole costs.

We observed 3070 restarts due to Spot instance terminations during this study; that is, restarts comprised about 15% of the overall job number. In the worst case, if an instance terminates just before writing a checkpoint, 1 ns of simulation time is lost. As we simulate 10 ns per job, an upper bound for the loss due to Spot instance terminations is about 1.5% of the total compute time. Spot instances get a signal two minutes before shutdown; thus, to completely avoid loss of compute time in the future, that signal could be used to trigger checkpoint writing in an improved simulation protocol. In addition to the performance and price evaluation, we have also validated correctness of the calculations against the previous computations.^69^ We used Student’s *t* test to compare free energy values calculated in the current work to those reported previously, ensuring that the results showed no statistically significant differences.

#### 4.4.2 Study 2: Focus on Cost-Efficiency

**Figure 11:**
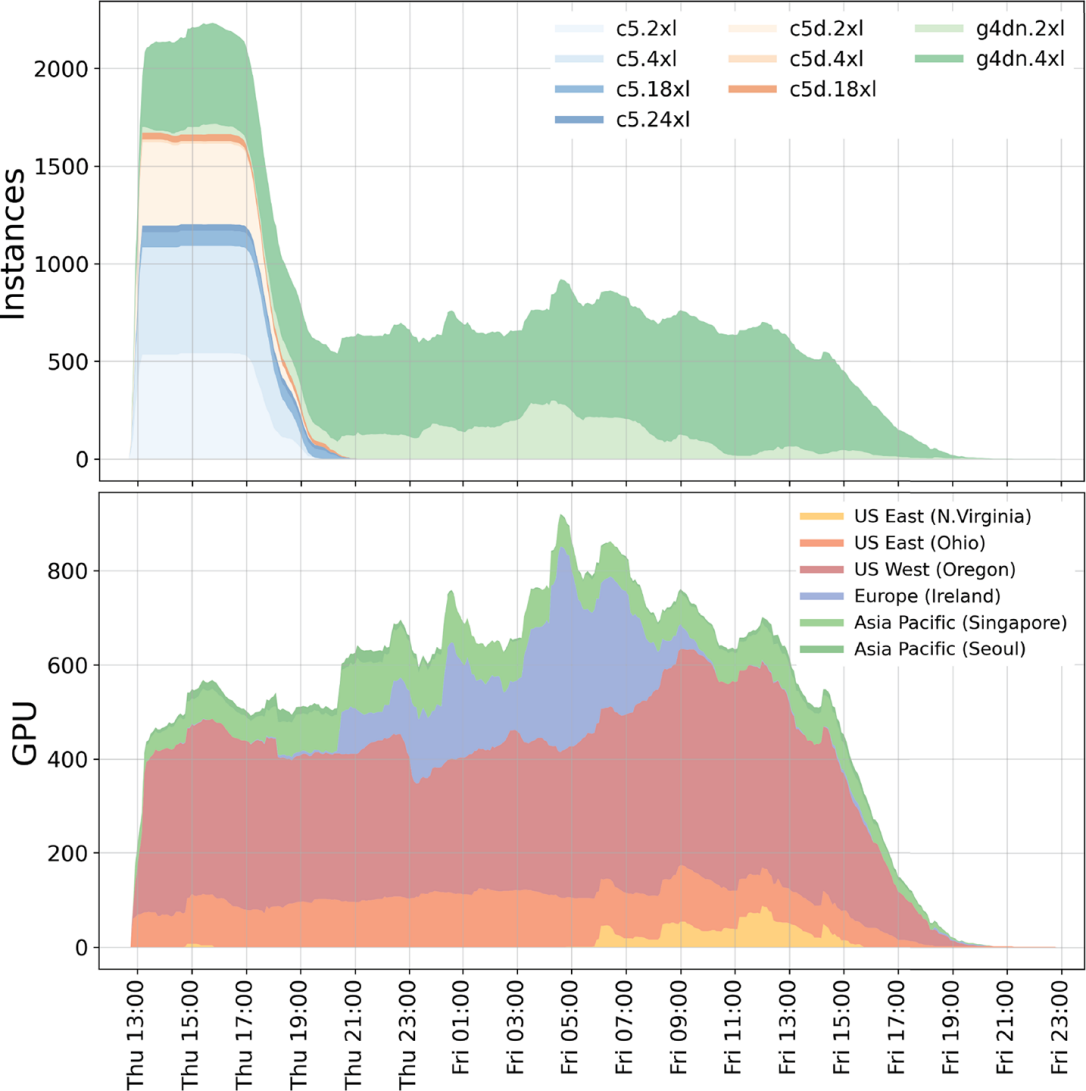
Usage of global compute resources for the second ligand screening study aimed at optimizing cost-efficiency. Top, allocated instance types over time; bottom, GPU instances allocated in the different regions.

Our second screening study aimed to further reduce the price tag by incorporating only the most cost-efficient instance types for the run. The second study used a slightly different and smaller data set (see Table 4) that required 6984 jobs to be run for 582 FE differences, or 70 μs of trajectory in total. The setup was as in the first study; however, we further excluded instances with low cost-efficiency. Most notably, we ran all the protein systems on cost-efficient GPU instances. The acquired resources over time for the second study are shown in Figure 11.

The vCPU usage peaked at slightly above 35 000 vCPUs at two hours into the second run (not shown), with on average 500 GPU instances in use over 26 h. About six hours after the submission of the ensemble the small ligand-in-water systems were finished (blue and orange areas in Figure 11, top). As our benchmarks on c5.2xl estimated a runtime of about five hours per system, we conclude that there were enough c5 Spot instances available to run each of the 3492 ligand jobs concurrently.

GPU instances are however running over a time span of about 30 h altogether, as apparently not enough g4dn Spot capacity was available to run all 3492 GPU jobs concurrently. From the lower panel of Figure 11 we see that at the time of submission, there was only g4dn capacity available in four regions, whereas the Ireland (blue) and North Virginia (yellow) regions provided g4dn instances only after several hours into the run. The large differences across regions underline that a multiregion approach is necessary for decent job throughput when limiting oneself to only a few instance types. The resulting costs of our second study were about $16 per FE difference and thus only about a third of what was achieved in the first study and in line with what is expected from the benchmarks on g4dn instances (Figure 8).

Both high-throughput ligand screening studies illustrate the flexibility of cloud computing for MD-based investigations: AWS can be used to scale up the computations to the extent of a large HPC facility but can also be used in a cost-efficient manner akin to a smaller in-house cluster. When aiming to minimize the time-to-solution, the 19 872 calculation jobs were finished in ∼2 days. This compares well to the timing in the recent report, where the Tier 2 Max Planck Supercomputer Raven (interim version, 480 Intel Xeon Cascade Lake-AP nodes with 96 cores, 192 threads) performed calculations of the same data set in ∼3 days.^69^ The cost-efficient usage of the cloud resources allowed reaching the cost of $16 for a free energy calculation. Cost-efficiency could be further optimized by also running the ligand-in-water simulations on the g4dn GPU instances (instead of using c5 instances), which would result in a cost of $14 per free energy difference, although g4dn capacity may then not be sufficient to run all simulations at once. In comparison to a GROMACS optimized in-house cluster of Intel E5-2630v4 10-core nodes and NVIDIA RTX 2080 GPU, this cost would be ∼$8.5, in agreement with the estimates of relative costs for a compute node analyzed in Figure 6.

## 5 Summarizing Discussion

Cloud computing has the potential to transform large-scale simulation projects. To date, computationally intensive projects, when assigned to busy on-premises clusters with limited computing capacity, may need weeks or months to be completed. In the cloud, though, the required processing power can be distributed among numerous compute centers around the globe. With the removal of the bottleneck of limited computing capacity, jobs that are independent of each other can run simultaneously in a high-throughput manner, thus reducing the time-to-solution to the runtime of the longest simulation of the ensemble. Such use cases that require very high peak performance over a short period of time can easily be met by cloud computing, while installing and operating a sufficiently large local cluster would be neither cost-effective nor feasible.

For the use case of MD-based high-throughput ligand screening we established a HyperBatch-based workflow that allows completing large-scale projects that would run for weeks on an on-premises cluster within 48 h or less in the cloud. Shortly after submitting 19 872 jobs, we acquired about 140 000 compute cores and 3000 GPUs in multiple regions around the globe. We demonstrated that the costs associated with such projects can be reduced about 9-fold compared to a naïve implementation: A job checkpoint-restart mechanism allowed using Spot instances instead of on-demand instances, which accounts for a 3-fold reduced price. Putting the benchmarked application performance in relation to the instance costs allowed selecting from a huge variety of available instance types the most cost-efficient ones only, thereby reducing the price tag by another factor of 3, albeit at the cost of a longer time-to-solution.

Whereas HyperBatch is geared toward speeding up HTC projects, we also investigated HPC strong scaling scenarios with a cloud-based HPC cluster. Cluster installation *via* ParallelCluster and software installation *via* Spack provided a straightforward and reproducible setup. Because of the possibility to automatically scale up (and down) the number of cluster nodes depending on whether there are jobs in the queue, there is virtually no waiting time for jobs in the queue. The breadth of readily available hardware that includes several architectures (Intel, AMD, ARM) in various sizes (regarding core count), accelerators like GPUs, and high-performance network if wanted, allows always picking the optimal hardware for the job at hand, in terms of a short time-to-solution or a high cost-efficiency. For GROMACS, we found that g4dn and g5 GPU instances offer the highest performance-to-price ratio, followed by hpc6a CPU instances, whereas instances with the fastest interconnect (c6i.32xl, hpc6a, and c5n.18xl) showed the best parallel scaling on up to 64 instances using 8192 vCPUs altogether for the largest benchmark system.

So how did, overall, cloud computing compare to a local cluster for our realistic test cases? For many cases, the extra flexibility offered by the cloud will certainly come at a cost higher than that of a local compute cluster. However, as our study shows, by aggressively tuning both alternatives toward cost-efficiency we are approaching a break even point, and the costs of cloud computing and on-premises computing become similar. In fact, an on-premises consumer-GPU cluster tailored toward GROMACS produces an MD trajectory at about 0.85× the costs of Spot GPU instances with similar performance.

We note that this outcome is also due to the fact that very specialized single software, GROMACS, was used; in contrast, if a wider variety of software has to run, the nodes can probably not be tuned that much and therefore will be less cost-efficient for a particular application. Just the use of a professional GPU instead of a consumer GPU will result in trajectory costs significantly higher than what can be achieved on an optimal Spot instance.

## 6 Conclusions

Cloud computing has traditionally been much more expensive than an on-premises cluster for the case in which continuous long-term computer performance is required. Here we have shown that this has changed, at least for the specialized, yet highly important application of drug design. We are now at a break even point, where the costs are the same, maintaining the great benefit of cloud computing to offer enormous flexibility and, if required, extremely short production times. We consider this a critical milestone for MD-based high-throughput computational drug design.

## Data and Software Availability

The input files for the benchmarks can be downloaded from https://www.mpinat.mpg.de/grubmueller/bench. A guide to build GROMACS on AWS is available here: https://gromacs-on-pcluster.workshop.aws. Free energy calculation benchmarks are available at http://pmx.mpibpc.mpg.de/aws.pl.
